# Effects of dietary FODMAP content on the faecal microbiome and gastrointestinal physiology in healthy adults: a randomised, controlled cross-over feeding study

**DOI:** 10.1017/S0007114525000868

**Published:** 2025-11-14

**Authors:** Nida Murtaza, Lyndal Collins, Chu K. Yao, Phoebe A. Thwaites, Patricia Veitch, Jane E. Varney, Paul A. Gill, Peter R. Gibson, Mark Morrison, Jane G. Muir

**Affiliations:** 1 Frazer Institute, Faculty of Medicine, The University of Queensland, Woolloongabba, QLD, Australia; 2 Department of Gastroenterology, School of Translational Medicine, Monash University and Alfred Health, Melbourne, Australia

**Keywords:** SCFA, Gastrointestinal transit, Gastrointestinal symptoms, Mycobiome, Polyols, Dietary intake, Psychological symptoms

## Abstract

The effect of dietary FODMAP (fermentable oligo-, di- and mono-saccharides and polyols) in healthy adults is poorly documented. This study compared the specific effects of low and moderate FODMAP intake (relative to typical intake) on the faecal microbiome, participant-reported outcomes and gastrointestinal physiology. In a single-blind cross-over study, twenty-five healthy participants were randomised to one of two provided diets, ‘low’ (LFD) <4 g/d or ‘moderate’ (MFD) 14–18 g/d, for 3 weeks each, with ≥ 2-week washout between. Endpoints were assessed in the last week of each diet. The faecal bacterial/archaeal and fungal communities were characterised by eighteen participants from whom high-quality DNA was extracted by 16S rRNA and internal transcribed spacer 2 (ITS2) profiling and metagenomic sequencing. There were no differences in gastrointestinal or behavioural symptoms (fatigue, depression, anxiety) or faecal characteristics and biochemistry (including SCFA). Mean colonic transit time (telemetry) was 23 (95 % CI: 15, 30) h with the MFD compared with 34 (24, 44) h with LFD (*n* 12; *P* = 0·009). Fungal diversity (richness) increased in response to MFD, but the bacterial richness was reduced, coincident with the expansion of the relative abundances of *Bifidobacterium*, *Anaerostipes* and *Eubacterium*. Metagenomic analysis showed expansion of polyol-utilising Bifidobacteria and *Anaerostipes* with MFD. In conclusion, short-term alterations of FODMAP intake are not associated with symptomatic, stool or behavioural manifestations in healthy adults, but remarkable shifts within the bacterial and mycobiome populations were observed. These findings emphasise the need to quantitatively assess all microbial domains and their interrelationships to improve understanding of the consequences of diet on gut function.

There is probably no more emotive subject than how one can use diet to improve and/or maintain good gut health and, in clinical medicine, improve outcomes in people with chronic conditions that include common gastrointestinal disorders, such as irritable bowel syndrome (IBS) or inflammatory bowel disease. Dietary manipulation can modulate intestinal injury and inflammation. For example, gluten induces injury in patients with coeliac disease and a gluten-free diet heals the injury^(1)^. Unfortunately, the popular press has portrayed the gluten-free diet as one that will improve the health of otherwise healthy people, but the scientific bases of such assertions are dubious^(2,3)^. Likewise, in patients with IBS, a condition that affects 4–10 % of populations across the world^(4)^, fermentable oligo-, di- and mono-saccharides and polyols (FODMAP), comprising mostly fructans, galacto-oligosaccharides, polyols, fructose in excess of glucose and lactose in those with hypolactasia, induce gut and systemic symptoms in the majority of patients^(5)^. Since the reduction of FODMAP intake ameliorates those symptoms^(6)^, a low FODMAP diet is now a recommended dietary approach in patients with IBS^(7,8)^. Since abdominal symptoms occur intermittently in the majority of the population^(9)^, there was a risk that healthy people who desire to be ‘healthier’ might take up a low FODMAP intake. Such a scenario was actively discouraged early in the development of the low FODMAP diet due to concerns that this may have detrimental effects on the gut^(10)^.

There are four main areas of concern regarding the effect of FODMAP on gut and general health. First, some FODMAP (such as fructose in excess of glucose) were first described to induce diarrhoea when consumed in high amounts^(11)^, leading to the concept that FODMAP were ‘natural laxatives’ and their restriction may lead to constipation. Indeed, many studies targeted patients with non-constipation IBS,^(12,13)^ and some reported poorer responses in patients with constipation predominance^(14,15)^. The reality is that feeding studies, in which variations of FODMAP intake from amounts in typical Australian diets to marked restriction, have reported no effect on faecal water content^(16)^ and more recent evaluation has reported similar value of restricting FODMAP irrespective of bowel habits^(17)^. However, the effects of such variations on gastrointestinal transit times have not been reported.

Second, oligosaccharides and other FODMAP may have injurious effects on the gut, as reviewed in detail^(5)^. In rodents fed large amounts of FODMAP, increased colonic permeability, mucosal inflammation, visceral hypersensitivity and bacterial translocation have been described^(18–22)^. In humans with IBS, inflammatory effects were observed in the rat colon of faecal supernatants from patients with IBS who were consuming a high-FODMAP diets^(23,24)^, and elevated circulating concentrations of inflammatory cytokines in patients with diarrhoea-predominant IBS reduced when FODMAP intake was reduced^(25)^. However, in healthy humans, the apparent effect of a high intake of FODMAP on intestinal permeability in patients with IBS was not reproduced^(26)^.

Third, considerable concern has been generated over the effect of reducing FODMAP intake on the gut microbiota. Many dietary FODMAP are non-digestible oligosaccharides, inulin and other short-chain carbohydrates that are also regarded as prebiotics, when defined as dietary components that selectively enhance the growth of select bacteria associated with health benefits^(27)^. Studies with prebiotics have generally involved adding inulin or non-digestible oligosaccharides as supplements to the diet, but few have addressed the intake and potential confounding effects of prebiotic FODMAP naturally occurring in food, including cereals fruit, vegetables and legumes. Most data have been derived from studying the effects on the microbiome of the reduction of FODMAP intake. Published data have been at times translated into scare-mongering that FODMAP restriction may be ‘wreaking havoc’ on the gut microbiota and may be detrimental to health^(28,29)^. In a meta-analysis of nine trials in 403 patients, diets very low in FODMAP have been associated with a reduction of the relative abundance of Bifidobacteria without consistent effects on other taxa in the faeces^(30)^, although one study showed it corrected dysbiosis in one-half of a cohort of patients with IBS^(31)^. The functional consequences of reduced FODMAP intake also might include reduction in delivery of SCFA, such as butyrate, to the colonic epithelium, and enhancement of protein fermentation believed to be detrimental to gut health. While a meta-analysis of published trials showed no consistent difference between their faecal concentrations with the low FODMAP and control diets^(30)^, reduced carbohydrate and enhanced protein fermentation were reported in sixty-three patients with IBS after 4 weeks of FODMAP restriction^(32)^. However, the specificity of such changes to FODMAP restriction was uncertain as the intake of long-chain fibres was not controlled.

Fourth, the relationship of FODMAP intake to mood disorders has received some attention. A high FODMAP diet-induced fatigue within 2 d in a cohort with IBS but not in healthy controls^(33)^. A high fructose intake was associated with mild depression in a cohort of young women with abdominal symptoms^(34,35)^. Paradoxically, prebiotic (FODMAP) supplements have improved mood disorders in some but not all studies of patients with mood disorders^(36)^.

Hence, the impact of altering the dietary intake of FODMAP in healthy people is not known. We hypothesised that short-term exposure to different levels of dietary FODMAP intake would have no discernible effect on the overt well-being of healthy adults but would influence the community structure of colonic bacteria and fungi. Hence, the study aimed to compare the short-term effects of two different levels of FODMAP intake in the setting of otherwise similar diets modelled on healthy diet guidelines – low FODMAP intake used in the first phase of the FODMAP dietary strategy in patients with IBS, and moderate FODMAP intake aimed to be above that of the typical Australian intake whereby potential prebiotic effects could be observed^(5)^. To do this, we performed a single-blinded, randomised, crossover feeding study in healthy adults and examined the effects on physiological/clinical endpoints ranging from subjective (gut symptoms, mood symptoms) to objective (transit time, regional pH, faecal SCFA and branched-chain fatty acids (BCFA) concentrations) and on the colonic (faecal) bacterial and fungal community.

## Experimental methods

### Ethical approval

Written, informed consent was obtained from all participants. The participants were not remunerated for their involvement. The protocol was approved by the Monash University Human Ethics Committee (MUHREC CF14/2904) and complied with the Declaration of Helsinki, with additional ratification for sample analyses obtained at the University of Queensland (UQHREC-2015000317). The protocol was registered at the Australian and New Zealand Clinical Trials Registry (ACTRN12617000205336) after the first patient was recruited, but there were no changes from the protocol approved by the Ethics Committee. The study report conforms with the CONSORT reporting guidelines for cross-over studies^(37)^.

### Participants

Healthy adult subjects (18–60 years of age) without known illness were recruited from advertising (Alfred Hospital, Monash FODMAP social media and School of Translational Medicine website) between August 2015 and January 2018. The first participant commenced in October, 2015 and the final participant completed the protocol in February, 2018. The participants had no pre-existing gastrointestinal disorders, were not currently consuming a restrictive diet (e.g., gluten-free diet), were not vegetarian or vegan (animal products were part of the diet), did not regularly suffer from gastrointestinal symptoms and were not lactose intolerant. They were excluded if they had been taking antibiotics, probiotics or supplemental prebiotics within 4 weeks, were on medication that is known to change intestinal transit (such as laxatives or hypomotility agents) and could not comprehend both verbal and written English.

### Protocol

**Figure 1.**
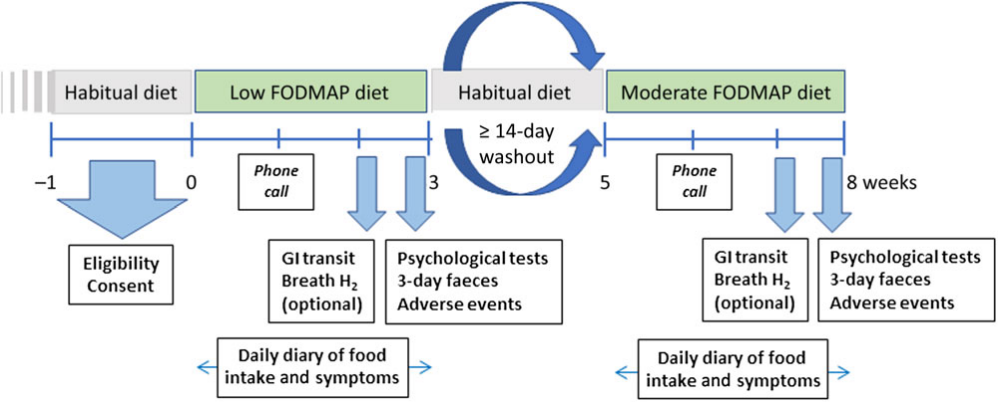
Study protocol. Abbreviation: GI, gastrointestinal.

The study protocol is illustrated in Figure 1. After the run-in evaluation assessment period, participants attended the laboratory for three visits with a one routine telephone check after 1 week. All assessments were performed by the study co-ordinator (L.C.). In a single-blinded, randomised, crossover design, participants were randomised according to a number list without blocking created with http://www.randomizer.org/ by the study coordinator (L.C.) to one of two dietary regimens for 3 weeks each. There was a washout period of at least 2 weeks before crossing over to the next diet in order to minimise carry-over effects. A cross-over design was used to minimise confounding from clinical and microbiological heterogeneity across individuals. A duration of 3 weeks was dictated by the practicality of a feeding study together with the knowledge that symptoms and microbial changes from altering FODMAP intake occur within that interval^(38)^. The wash-out period was similar to that utilised previously^(38)^.

The participants completed daily diaries regarding food intake and gastrointestinal symptoms throughout each dietary intervention. As an optional extra visit during the 2 weeks of each diet, participants were provided breath bags and instructed on taking hourly breath samples at home over a 12-hour period. The bags were returned for evaluation at the next visit. On the first day of week 3 of each diet, the participant attended the laboratory after an overnight fast to swallow a telemetric capsule. Over the last 3 days of each dietary period, faeces were collected and returned on the last day, when behavioural questionnaires were repeated and adverse events documented. Height and weight were measured by the study dietitian at the initial assessment and the end of each dietary period with the subject similarly dressed. The interventions and the clinical and gastrointestinal assessments were performed at the Monash University facility at the Alfred Hospital, Melbourne, Victoria. The microbiological analyses were carried out at the Frazer Institute at Translational Research Institute, Woolloongabba, Queensland. The clinical co-ordinator was not blinded, but physiological, laboratory and microbiological analyses were all performed by personnel blinded to the intervention with all information being identified by a personal identification number only.

### Diets

The intervention diets were designed to vary only in terms of total FODMAP content. Since lactose is only a FODMAP in the presence of hypolactasia, lactose content was not included when calculating total FODMAP content. The two diets were arbitrarily defined as: (a) ‘*Low’ FODMAP diet* (designated ‘LFD’) containing < 4 g/d of FODMAP as might be expected during a low FODMAP restrictive phase^(5,38,39)^ and (b) ‘*Moderate’ FODMAP diet* (designated ‘MFD’) containing approximately 8 g of FODMAP oligosaccharides and 14–18 g/d of total FODMAP, representing a small increase in FODMAP intake to that of the average Australian diet^(5,40)^. To assist with dietary protocol compliance, we provided approximately 80 % of participants with total energy requirements through prepared meals. These meals, including breakfast, lunch, dinner and some snacks, were supplied frozen, along with detailed reheating instructions. However, to incorporate fresh foods into the diets of participants, we provided a specific list of items adjusted to each group – one for the LFD and another for the MFD – to purchase themselves. These included fresh salad vegetables, fruits, dairy products (such as milk, cheese and yoghurt) and beverages (including juices, tea, coffee). These food items are best consumed fresh, as freezing would compromise their quality and palatability. The provided food was prepared in commercial kitchens at Monash University under the supervision of a research chef (P.V.). The meals were blinded to the participants as to which diet they belonged by labelling only by number. The meals were vacuum-packed and frozen until delivered to the recipient’s home address. Menu planning was guided by and complied with the Australian Dietary Guidelines of the National Health and Medical Research Council of Australia^(41)^. Lean red meat was supplied by Meat & Livestock Australia, and the grain ingredients were supplied by the Grains & Legumes Nutrition Council. An example of a 1-day meal plan for the diets is shown in online Supplementary Table 1. Both diets were introduced with graded increases in the FODMAP-rich foods to reach the targeted intake over the first few days in order to avoid bloating and abdominal discomfort.

### Assessment of dietary intake

From daily food intake diary entries (using household serves and weighed food measurements including details about ingredients, brands of food and cooking methods), nutritional composition was calculated using nutrition analysis software, Foodworks X7 (Xyris Software; Brisbane; Australia), containing compositional data of short-chain carbohydrates from the Monash database and resistant starch from published materials^(42–45)^. Additional evaluation of ultra-processed foods was performed using the NOVA classification^(46)^.

### Assessment of dietary adherence

Adherence to the diet was assessed according to actual food intake during the interventions via direct questioning by a dietitian (L.C.) and daily diary entries for the interventional periods and was arbitrarily rated according to the proportion of supplied meals consumed as ‘excellent’ if >80 % were consumed, ‘good’ for 60–80 % and ‘poor’ for < 59 %. In addition, hourly breath samples were collected over a 12-hour period at the end of the second week of the diets in a sub-group. The purpose of this was to provide additional support that the diets were being consumed as stated since the amount of intestinal fermentation, measured by hydrogen and methane excretion, should be different between the two diets. Since this was only supportive evidence of adherence and since there was considerable protocol burden for the participants, it was offered as an ‘optional’ test. They were considered ‘hydrogen-producers’ and/or ‘methane-producers’ if at least one reading over the day was > 5 ppm, respectively. Areas under the curve over 12 h were calculated and compared between the diets. The methodology used and calculations made were as previously described^(33)^.

### Analytical methods

#### Gastrointestinal symptoms

These were assessed via daily diary cards with 100-mm visual analogue scales to score overall and individual gastrointestinal symptoms including bloating, wind, abdominal pain and fatigue, as previously applied^(38)^. The frequency of bowel actions was noted in the diary cards.

#### Regional gastrointestinal transit times and pH

In the last week of each 3-week dietary period, participants were invited to ingest a telemetric wireless motility capsule (SmartPill®, Medtronic, Dublin, Ireland) that transmits data related to pH, temperature and pressure every 5 min to a wearable data receiver^(47,48)^. Information was downloaded and interpreted using dedicated software (MotiliGI, version 3.0, Medtronic). After an overnight fast, participants consumed their allocated breakfast, swallowed the capsule and then were permitted water only for 6 h, after which normal intake was resumed. Anatomical landmarks were identified by changes in temperature and pH profiles along the gastrointestinal tract, enabling calculation of gastric emptying time, small bowel transit time, colonic transit time and whole gut transit time, as previously described^(48,49)^. Gastric emptying times greater than six hours were not included in the analysis. Luminal pH was expressed as the average across the small bowel and the average for each quartile of colonic transit.

#### Behavioural measures

During the last week of each dietary arm, participants completed three questionnaires – the State-Trait-Personality Inventory^(50)^, the Abbreviated Depression Anxiety Severity Scale (DASS-21)^(51)^ and the Daily Fatigue Impact Scale^(52)^. Details of the scales and their interpretation are shown in the online Supplementary Information.

#### Faecal indices

Faeces were collected for the last 3 d of each dietary period. All faeces were passed into plastic containers with care to avoid urine contamination. These containers were immediately placed into portable –20°C freezers (supplied to the participants). After delivery to the laboratory, samples were thawed and pooled, weighed (total output), and then homogenised from which multiple aliquots were frozen and stored at –40°C before analysis within 9 months of collection. Water content was measured in an aliquot by freeze-drying (Operon, Thermo Fisher Scientific Australia; Scoresby, Victoria, Australia). pH was measured with a calibrated pH probe (Five-Go pH meter & pH electrode LE427, Mettler-Toledo; Schwereznbach; Switzerland) with the sample at 25°C in a water bath. SCFA and BCFA were measured in triplicate by gas chromatography as previously described in detail^(53)^. The concentrations of phenol and p-cresol were measured by HPLC^(54)^. Calprotectin was measured by ELISA (Bühlmann Laboratories, Schönenbuch, Switzerland) as per the manufacturer’s instructions.

### Faecal microbiota analyses

These procedures are described in detail in the online Supplementary methods. Briefly, total DNA was extracted from subsamples of the preserved stool samples using a repeated bead-beating lysis protocol and purified by an automated column-based purification system^(55,56)^. PCR amplification reactions with primers that selectively target either the V6–V8 hypervariable regions of Bacteria/Archaea 16S rRNA, or the Fungal ITS-2 region^(57,58)^ were used. A third subsample of stool DNA was used to construct libraries for shotgun metagenomic sequencing. Excepting the stool DNA extractions, all the protocols and sequencing platforms used were provided by the University of Queensland’s Australian Centre for Ecogenomics (www.ecogenomics.org). The resulting raw datasets were processed to trim and recover the high-quality reads using established protocols^(57)^(online Supplementary methods), and taxonomic assignments of the PCR amplicons representing bacteria/archaea and fungi were made using the SILVA and UNITE databases, respectively^(59,60)^. The metagenomic data were analysed using the HUMANn2 work package^(61)^ and also processed for the recovery of metagenome-assembled genomes (MAG) using MetaBAT^(62)^ and uploaded to the Pathosystems Resource Integration Center (PATRIC) work package for taxonomic and functional characterisation^(63,64)^.

### Statistical analyses and justification of sample size

Utilising a crossover design, we determined that a sample size of 24 would be sufficient to achieve an 80 % power at 5 % significance for a one-tail test. This estimation was derived from a prior study^(38,65)^, where changes in clinical and physiological endpoints were assessed following manipulation of dietary FODMAP intake (low *v*. moderate) among individuals with IBS. All analyses were performed per protocol. Statistical analyses for biochemical, physiological and clinical data were performed using GraphPad Prism (version 9.2.0). Summary data were expressed by median (interquartile range) or mean (95 % CI) depending upon the distribution of the data. The measured indices were compared between the diets using repeated measures, a paired *t* test or Wilcoxon signed-rank test. The statistical significance level for clinical and physiological endpoints was set at 0·05, except where Bonferroni’s correction for multiple comparisons was made.

The microbiota taxonomic count data were first normalised by square-root transformation, then subjected to repeated-measures statistical analyses via mixed effect linear regression analysis in Calypso version 8.18^(66)^. The data were also subjected to sparse partial least squares discriminant analysis (sPLS-DA) using the MixOmics mixMC: multivariate data analysis framework^(67)^ to identify the taxonomic and functional features discriminatory for LFD and MFD groups. Spearman’s correlations were also calculated from the non-normally distributed data, the correlation plots were made using the *corrplot* package and the adjusted *P* values were calculated using the p-adjust function in R. The threshold for statistical significance was set at *P* ≤ 0·05 for all the analyses. The corrections for multiple testing by false discovery rate (FDR) are also reported and categorised as significant (FDR < 0·05), moderate (FDR < 0·3) or large (FDR > 0·3). Only those differences with FDR < 0·05 were used for Spearman’s correlation analyses.

## Results

### Participants

**Figure 2.**
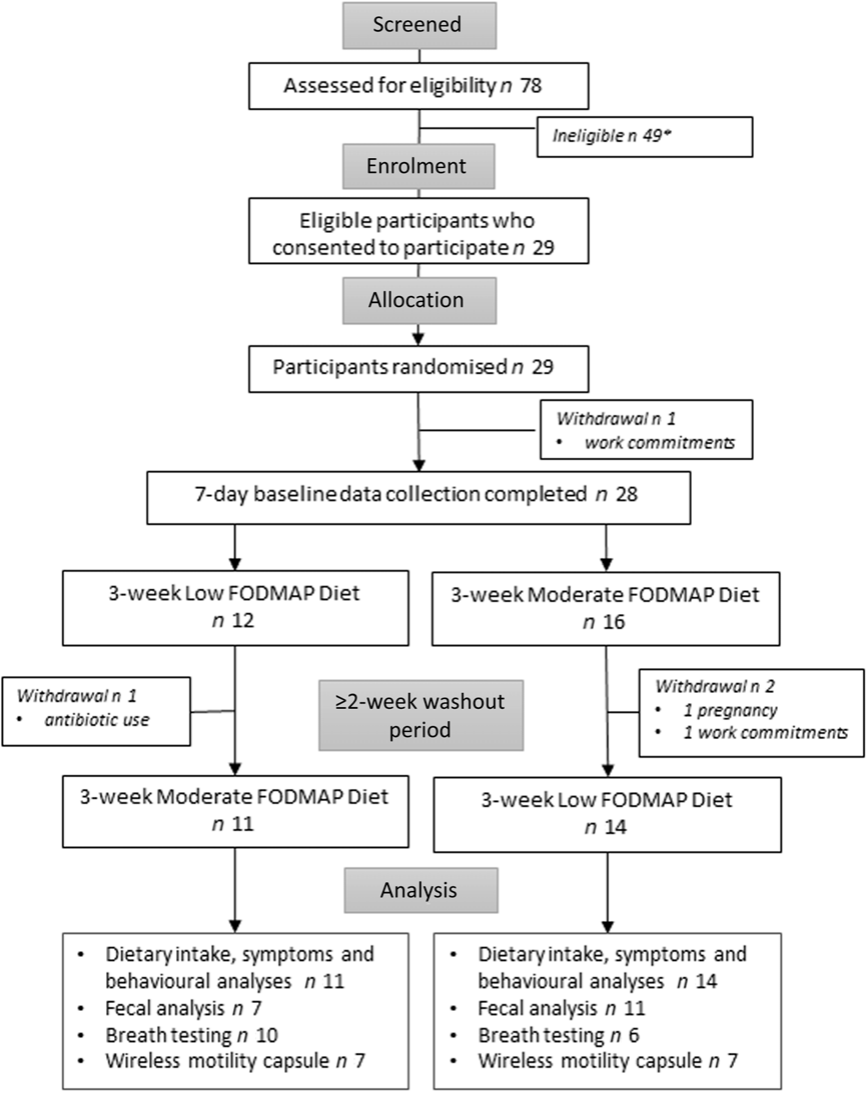
CONSORT diagram of patient flow and analysis. * not interested, recent antibiotic use, food allergies (such as nuts, which were used in the study diets), unable to commit to the time required, breastfeeding or not comfortable collecting biohazards.

Of twenty-nine recruits, four withdrew, as shown in Figure 2. Thus, data from twenty-five participants (16 female) with a mean age of 43 (95 % CI 36, 49) years and BMI 25·1 (23·3, 26·8) kg/m^2^ were included in the dietary, symptom and behavioural analyses. Eighteen participants completed a minimum of 3-day faecal collection at the end of each dietary period together with high-quality DNA extraction enabling microbiological analysis. Sixteen elected to perform breath tests. Fourteen participants had technically successful wireless motility capsule studies for both arms of the study.

### Dietary intake

Analysis of dietary intake according to the food intake diaries is shown in Table 1. There were differences for total FODMAP (*P* < 0·001; paired *t* tests), specifically a 3·5-fold increase in intake of oligosaccharides (*P* < 0·001), a seven- and six-fold increase in total polyols (*P* < 0·001) and excess fructose (*P* < 0·001) respectively, in the moderate compared with those in the low FODMAP diet. Dietary fibre intake (not including FODMAP) was a mean 5·5 g/d greater in the MFD compared with that in the LFD. Numerically small but statistically significant differences between the two diets were also detected for the intake of total and saturated fats.

**Table 1.** Actual daily dietary intake of the twenty-five participants according to 7-day food diaries during the two dietary interventions shown as the mean (95 % CI)

Food component		Low FODMAP diet		Moderate FODMAP diet		Differences		*P*-value^*^
		Mean	95 % CI	Mean	95 % CI	Mean	95 % CI	
Energy (MJ)		8·4	7·8, 9·0	8·5	7·8, 9·1	–0·05	–0·4, 0·3	0·71
Protein (g)		100·0	93·5, 106·5	100·0	93·0, 107·5	–0·2	–3·8, 3·4	0·90
Fat (g)	Total	77·7	70·3, 85·1	72·4	65·9, 78·9	5·3	2·0, 8·6	0·003
	Saturated	26·9	24·2, 29·7	24·1	21·9, 26·4	2·8	1·3, 4·4	0·001
Carbohydrates (g)		208·2	193·7, 222·7	221·9	205·4, 238·4	–13·7	–22, 5·3	0·002
Non-digestible polysaccharides	Dietary fibre	29·6	27·5, 31·7	35·1	32·7, 37·5	–5·5	–6·6, 4·4	< 0·001
	Resistant starch	1·8	1·6, 2·0	2·0	1·8, 2·2	–0·2	–0·4, −0·04	0·02
FODMAP (g)	Total^†^	3·5	3·2, 3·9	16·5	14·8, 18·1	–12·9	–14·4, −11·5	< 0·001
	Oligosaccharides	1·9	1·6, 2·1	7·0	6·4, 7·6	–5·1	–5·6, −4·7	< 0·001
	Fructans	1·6	1·3, 1·8	5·3	4·8, 5·8	–3·7	–4·1, −3·4	< 0·001
	Galacto-oligosaccharides	0·3	0·3, 0·3	1·8	1·6, 1·9	–1·5	–1·6, −1·3	< 0·001
	Excess fructose	1·2	1·0, 1·4	6·2	5·3, 7·1	–5·0	–5·8, −4·2	< 0·001
	Polyols	0·5	0·4, 0·6	3·3	2·9, 3·8	–2·8	–3·2, −2·4	< 0·001
	Sorbitol	0·3	0·3, 0·4	1·9	1·6, 2·2	–1·6	–1·8, −1·3	< 0·001
	Mannitol	0·2	0·1, 0·2	1·4	1·3, 1·6	–1·3	–1·5, −1·1	< 0·001
	Lactose	20·6	17·9, 23·2	21·1	18·2, 24·0	–0·5	–1·9, −0·8	0·42

FODMAP, fermentable oligo-, di- and mono-saccharides and polyol.

* Paired *t* test; the *P*-value considered statistically significant was set at 0·003 (Bonferroni correction).

† Total FODMAP intake was calculated as the sum of oligosaccharides, excess fructose and polyols.

Adherence to the intervention diets was judged as excellent for twenty-three participants and good for two on the basis of reported food intake. Increased breath hydrogen was observed in association with the MFD compared with the LFD in the subgroup who accepted the optional offer to undertake breath tests and were hydrogen producers. Thus, the area under the curve for hydrogen (*n* 15) was consistently greater at a mean of 15 296 (95 % CI 9678, 20 914) ppm.12 h during the MFD than 7748 (4911, 10 585) ppm.12 h during the LFD (*P* < 0·001). Similarly, mean breath methane in those who also produced methane was greater with the MFD at 36 445 (24 180, 48 710) ppm.12 h compared with 18 635 (12 340, 24 930) ppm.12 h during the LFD (*P* < 0·001, online Supplementary Figure 2). Both diets were well tolerated with no adverse events related to the interventions.

### Clinical and behavioural measures

These measures are presented in Table 2. The weight of the participants was stable throughout the study. Gastrointestinal symptoms were reported at very low levels (< 20 mm of the 100-mm visual analogue scale) during the baseline period, and no changes were noted with either the LFD or MFD. Scores from two different tests of psychological status (anxiety, depression and stress) and a questionnaire to assess fatigue also indicated no differences between the paired results during the LFD and MFD. No adverse events were reported during the dietary periods.

**Table 2.** Clinical and behavioural measures in twenty-five participants during the interventional dietary periods. Data are shown as median (interquartile range)

Measure		Low FODMAP diet		Moderate FODMAP diet		*P*-value^*^
		Median	IQR	Median	IQR	
Gastrointestinal symptoms (100-mm visual analogue scale), mm	Overall	7·0	2·0–11·5	7·0	3·0–12·0	0·93
	Abdominal pain	5·0	2·0–7·5	5·0	2·5–9·0	0·91
	Bloating	6·0	2·5–14·0	4·0	2·0–15·0	0·76
	Wind (flatus)	8·0	3·5–16·0	10·0	3·5–19·0	0·48
	Nausea	2·0	0–4·5	2·0	0–6·0	0·68
	Fatigue	6·0	2·0–17·0	9·0	2·0–14·5	0·84
State-Trait-Personality Inventory (STPI)^†^	State anxiety	34	30–37	34	33–37	0·24
	State depression	37	34–39	38	35–40	0·26
	Trait anxiety	37	34–39	36	34–38	0·89
	Trait depression	38	37–40	38	37–40	0·10
Abbreviated Depression Anxiety Severity Scale (DASS-21)^‡^	Depression	2	0–4	2	0–4	0·81
	Anxiety	0	0–2	1	0–2	0·81
	Stress	4	0–10	2	0–8	0·28
Fatigue (D-FIS)^§^	Overall score	3	0–7·5	3	0–6	0·66

FODMAP, fermentable oligo-, di- and mono-saccharides and polyol.

* Wilcoxon signed ranked test.

† STPI is an eighty-item self-report questionnaire, with eight ten-item scales. State items are used to assess the current emotional state and are rated on a four-point intensity scale, where 1 = not at all; and 4 = very much so. Trait items assess emotional disposition and are rated on a four-point intensity scale where 1 = almost never and 4 = almost always. The range of possible scores for each subscale can vary from a minimum of 10 to a maximum of 40.

‡ DASS-21 comprises twenty-one items. Responses are recorded via a four-point severity scale, with total scores for each domain derived by summing the responses for their respective items. Higher scores represent greater severity; the maximum possible score for each domain is 21.

§ D-FIS is a forty-item scale that encompasses physical (ten items), cognitive (ten items) and psychosocial domains (twenty items). Higher scores represent a greater impact of fatigue.

### Gastrointestinal transit times and regional pH

**Figure 3.**
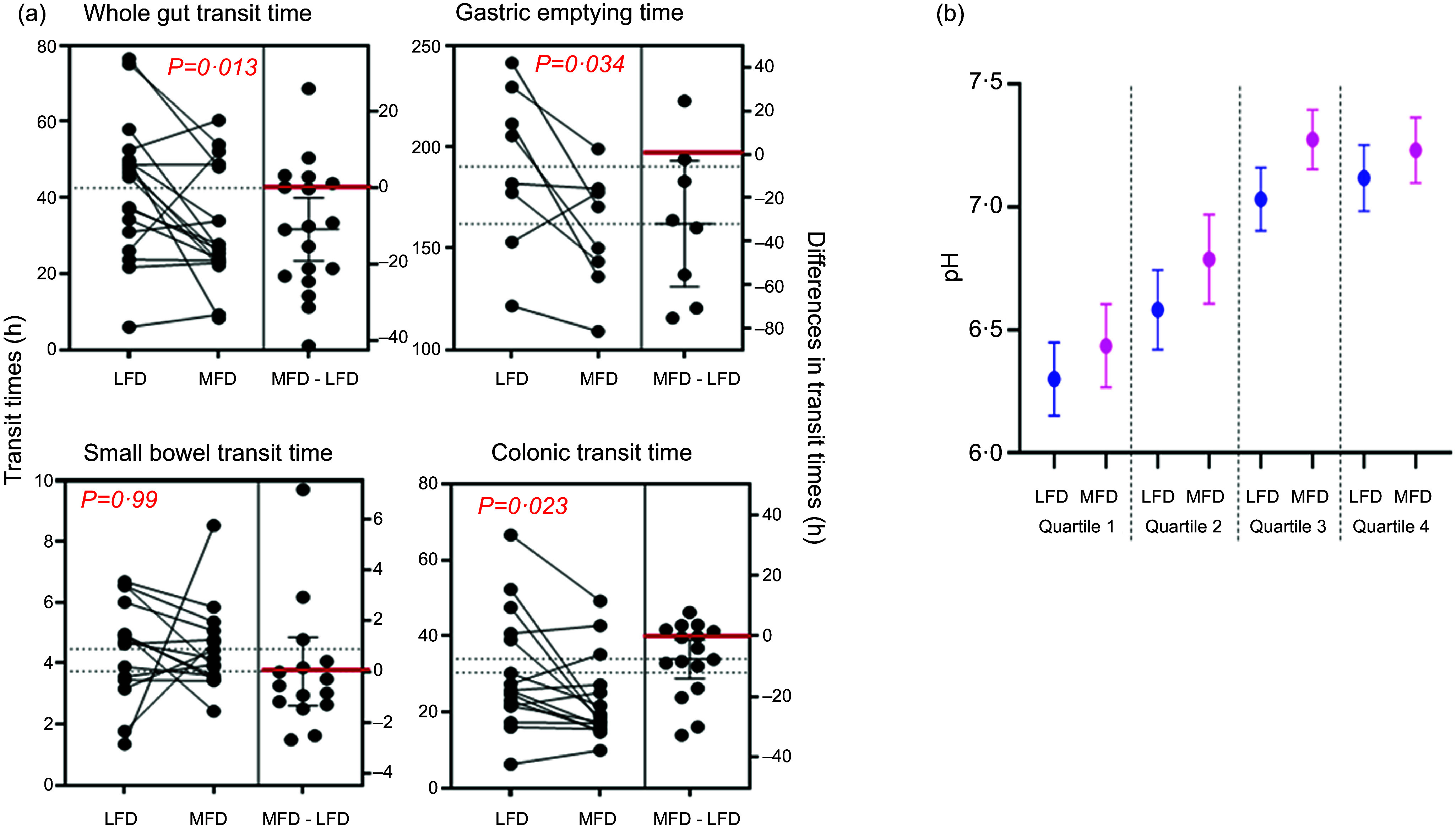
Results from the wireless motility capsule. (a) Regional gastrointestinal transit times and their mean differences during the low FODMAP diet (LFD) and moderate FODMAP diet (MFD). The red line represents no differences. Statistical results from paired *t* tests are shown in the graphs. (b) Luminal pH in quartiles of colonic transit during the diets. Results are shown as mean and 95 % CI. No statistically significant differences were observed between the diets in the quartiles (paired *t* tests) or across all quartiles (repeated-measures ANOVA).

As shown in Figure 3, whole-gut transit times were shorter with the MFD than with the LFD (*n* 14; *P =* 0·018). This was reflected in faster gastric emptying (*n* 8; *P =* 0·03) but not small bowel transit times (*n* 12). Of twelve participants with evaluable data, colonic transit time was 34 (24, 44) h with the LFD compared with 23 (15, 30) h with the MFD (*P* = 0·009). Two participants had slower transit in the colon with the MFD and both were methane producers. However, three other methane producers had faster transit with the MFD compared with the LFD.

Across the small bowel, average luminal pH was 7·2 (6·9, 7·6) with the MFD, which was similar to 7·0 (6·7, 7·3) with the LFD (*n* 12; *P* = 0·41). Luminal pH in each quartile of colonic transit during the interventional dietary periods increased distally, but no differences in this pattern, nor in the paired pH in each quartile were observed (Figure 3(b)).

### Faecal measures

Summary data on faecal measures are shown in Table 3. Daily faecal output and number of bowel actions over 3 d, as well as faecal water content, pH, and calprotectin concentrations were not statistically significantly different during the LFD and MFD. The concentrations, daily excretion and relative proportions of the major SCFA (acetate, propionate and butyrate) were not statistically different between diets, and there were no carry-over effects from one interventional diet to the other evident (data not shown). Furthermore, there were no statistically significant differences between diets for the faecal concentrations of caproate and valerate, of BCFA, isobutyrate and isovalerate, and of total phenols and its major (> 95 %) component, *p*-cresol. Likewise, the ratio of SCFA:BCFA was not different.

**Table 3.** Faecal measures during the interventional dietary periods in eighteen participants who provided complete samples. Data are shown as mean (95 % CI) and statistically compared between diets using paired *t* test, except where denoted

Measure		Low FODMAP diet		Moderate FODMAP diet		*P*-value
		Mean	95 % CI	Mean	95 % CI	
72-hour output (g/d)		185	139, 265	188	145, 273	0·05
		Median	IQR	Median	IQR	
Number of bowel actions in 72 h^*^		5	3–5	5	3–7	> 0·30^†^
		Mean	95 % CI	Mean	95 % CI	
Water content (%)		70	67, 74	72	69, 74	0·15
pH		6·5	6·4, 6·6	6·5	6·4, 6·6	0·26
SCFA, concentration, µmol/g	Acetate	57	48, 65	60	51, 70	> 0·30
	Propionate	17	15, 19	16	14, 19	> 0·30
	Butyrate	17	15, 19	17	15, 20	> 0·30
	Valerate	2·1	1·8, 2·4	1·9	1·6, 2·3	> 0·30
	Caproate	0·7	0·4, 1·0	0·6	0·4, 0·9	0·14
Proportion of total SCFA	Acetate	56	(44, 68)%	57	(47, 67)%	> 0·30
	Propionate	18	(10, 26)%	16	(10, 22)%	> 0·30
	Butyrate	19	(11, 27)%	18	12, 24)%	> 0·30
Branched-chain fatty acids (BCFA), concentration, µmol/g	Isobutyrate	1·9	1·7, 2·2	1·8	1·4, 2·1	0·15
	Isovalerate	2·8	2·4, 3·2	2·5	2·0, 3·0	0·06
SCFA:BCFA		22	17, 27	28	19, 37	0·12
Phenols, concentration, µg/g	Total	51	37, 66	43	27, 59	0·11
	Phenol	4·0	1·8, 6·1	3·8	1·2, 6·4	0·14
	*p*-Cresol	47	33, 62	40	23, 56	0·13
Calprotectin, µg/g		21	12, 28	17	10, 25	0·07

FODMAP, fermentable oligo-, di- and mono-saccharides and polyol.

* Median (IQR).

† Wilcoxon signed rank test.

### Measures of the faecal microbiota

#### Effect on bacterial richness and alpha (within-sample) microbial diversity

**Figure 4.**
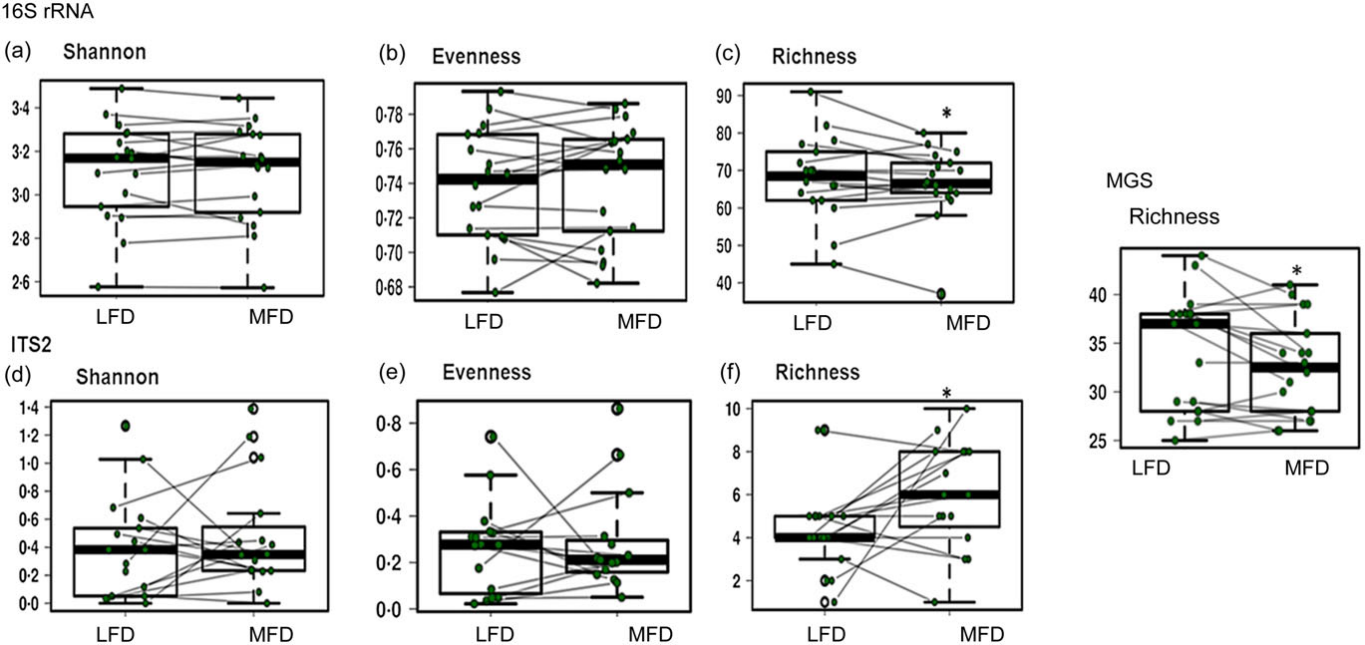
Measures of the alpha (within sample) diversity for the prokaryote (16S rRNA and MGS) and fungal (ITS2) communities recovered from the stool samples of healthy adults following their consumption of the low FODMAP (LFD) or moderate FODMAP diet (MFD). As described in the Results, only the changes in Richness scores in response to the MFD were deemed to be statistically significant.

The results of these analyses are presented in Figure 4. There were no significant differences between the Shannon diversity metrics calculated for both the Bacteria/Archaea and Fungal Domains following the LFD and MFD (Figure 4(a) and (d)). Given that the Shannon diversity metric is a composite measure derived from the microbial richness and evenness within individual samples, the richness and evenness scores were also examined separately. These analyses showed that while the evenness scores remained similar (Figure 4(b) and (e)), the alterations in the richness scores in response to the MFD for Bacteria/Archaea and Fungi were both statistically significant but in opposite directions. Whereas the richness scores for the Bacteria/Archaea were reduced with the MFD (*P* = 0·052, Figure 4(c)), the fungal richness scores increased with the MFD (*P* = 0·03, Figure 4(f)). The taxonomy-based assessment of the shotgun metagenomic sequencing (MGS) data also showed that bacterial richness was reduced in response to the MFD (*P* = 0·014, Figure 4) but with limited impact on the evenness and Shannon diversity metrics of the Bacterial/Archaeal communities with the LFD and MFD (data not shown).

**Figure 5.**
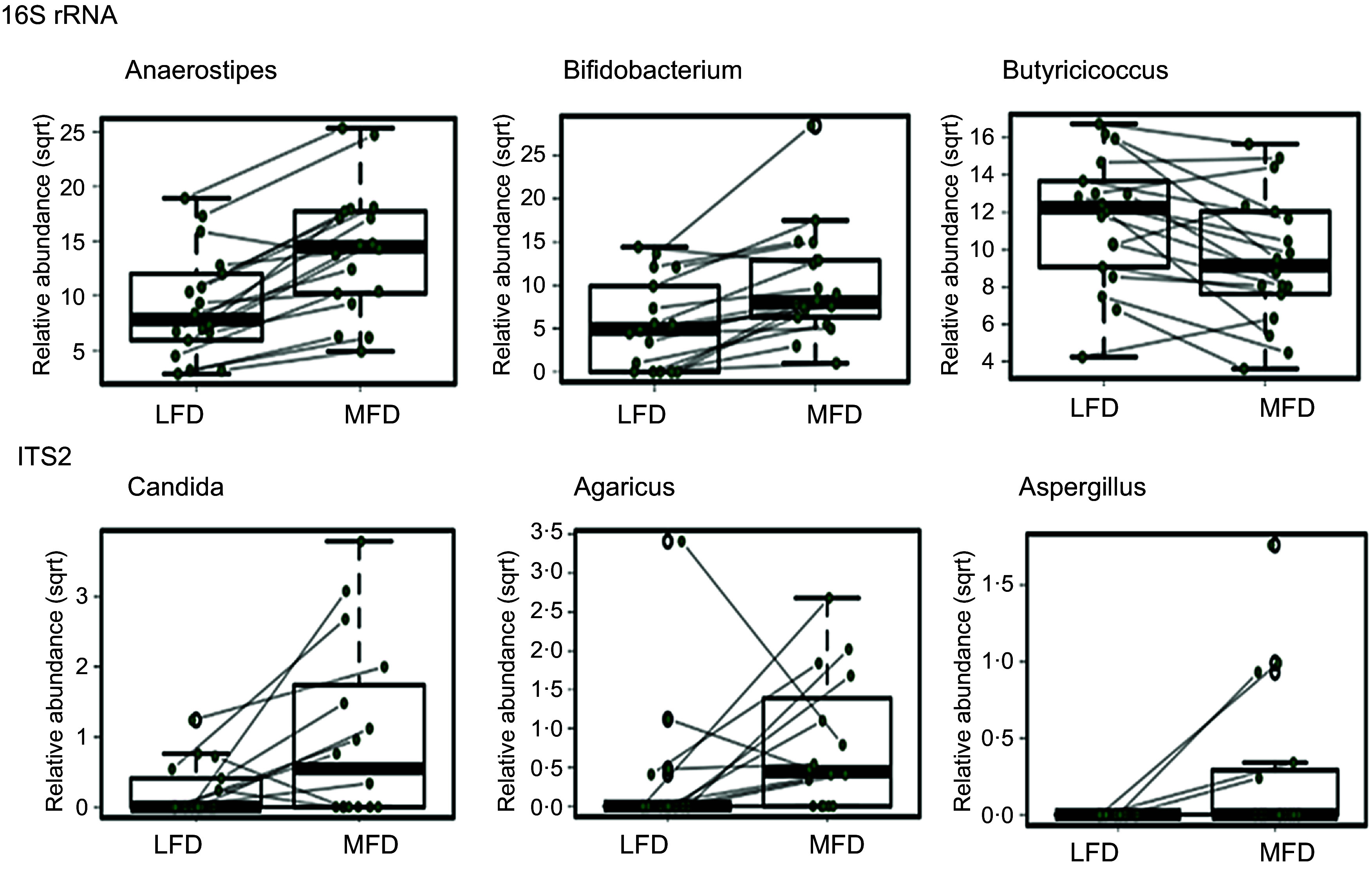
Mixed effect linear regression of key changes in select bacterial (16S rRNA) and fungal (ITS2) taxa between consumption of the low FODMAP diet (LFD) and moderate FODMAP diet (MFD). Data collected from the same subject are connected by the lines. As described in the Results, these differences between diets for were all found to be statistically significant and remained so upon tests for multiplicity (FDR correction).

Figure 5 shows the reductions in Bacterial species counts (richness) appeared to be in response to an expansion of the relative abundances of *Anaerostipes* (*P* < 0·001, FDR < 0·001) and *Bifidobacterium* (*P* < 0·001, FDR < 0·001), and there was also a concurrent reduction in the relative abundance of *Butyricoccus* (*P* = 0·001, FDR = 0·022) in response to the MFD (Figure 5). The mixed effect linear regression analysis of the fungal ITS2 data also identified differences between the diets, with greater relative abundances of *Candida* (*P* = 0·003, FDR = 0·087) and *Aspergillus* (*P* = 0·027, FDR = 0·2, Figure 5) in response to the MFD. The relative abundance of reads assigned to the genus *Agaricus* was also greater in response to MFD (*P* = 0·033, FDR = 0·2) and are deemed to be of dietary origin.

Based on these results, the amplicon datasets were also examined using sPLS-DA to identify other microbial taxa that were discriminatory between the communities after consumption of either the LFD or MFD. The sPLS-DA analyses suggested that, in addition to *Anaerostipes* and *Bifidobacterium, Prevotella 7* and members of *Lachnospiraceae* ND3007 group discriminated between the Bacteria/Archaea communities in response to the MFD diet; and *Haemophilus* spp. was discriminatory of the communities observed following consumption of the LFD (online Supplementary Figure 2). The sPLS-DA analysis of the ITS2-derived profiles also supported the finding that *Aspergillus* are discriminatory of the mycobiome with the MFD, whereas the genera *Byssochlamys*, *Meira* and *Leucosporidium* were discriminatory of the LFD (online Supplementary Figure 2(b)).

The MGS data metrics from the samples analysed in this study are shown in online Supplementary Table 2. The Bowtie2 alignment against the human hg19 database removed ∼1 % of the reads, and the range of paired-end reads remaining was similar for each subject and the two dietary groups. Notably, and unlike the PCR amplicon datasets, the archaeal and fungal populations were either underrepresented or not detectable, respectively, within our MGS datasets, most likely reflective of their relatively low abundance in the stool.

**Figure 6.**
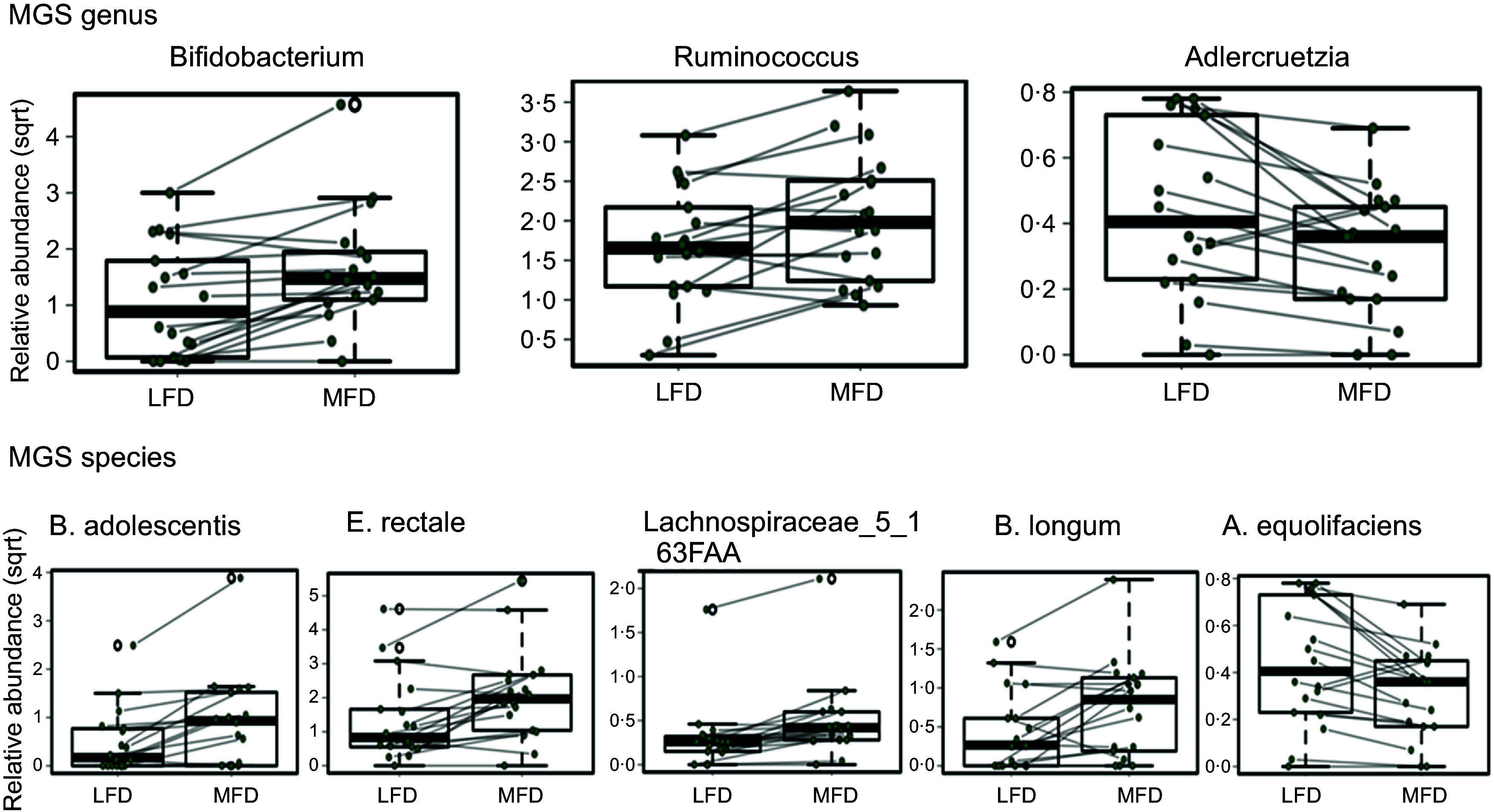
The key changes in select bacterial taxa detected by mixed effect linear regression analysis of the metagenomic sequence (MGS) datasets at the genus and species level following consumption of the low FODMAP diet (LFD) and moderate FODMAP diet (MFD). Data collected from the same subject are connected by the lines. As described in the Results, these differences between diets for were all found to be statistically significant (*P* < 0·05) but with variable strength upon tests for multiplicity (FDR correction).

Figure 6 shows the key changes in bacterial taxa at the genus and species levels detected via mixed effect linear regression analysis. Again, the genus *Bifidobacterium* was significantly increased in response to MFD and remained so after FDR correction (*P* < 0·001, FDR = 0·014). The significant increase in the genus *Ruminococcus* (*P* = 0·018) and the decrease in the genus *Adlercruetzia* (*P* = 0·02) however were moderated after multiplicity testing (FDR = 0·7 and 0·3, respectively). At the species level *Eubacterium rectale* (*P* < 0·01), *Lachnospiraceae* bacterium (*P* < 0·01) and *Bifidobacterium longum* (*P* = 0·03) were all significantly increased, and the relative abundance of *Adlercruetzia equolifaciens* (*P* = 0·02) was decreased after consumption of the MFD. However, the significance of these species-level differences was also moderated after multiplicity testing (FDR = 0·3, 0·3, 0·7 and 1·0, respectively). In light of this, the MGS data were subjected to sPLS-DA and here, relative abundances of *Bifidobacterium longum*, *Lachnospiraceae_*5_1_63 FAA, and *Eubacterium rectale* were all discriminatory of the MFD diet, while *Alistipes shahii* and *Adlercruetzia equolifaciens* were more abundant and discriminatory of the LFD diet (online Supplementary Figure 3(c)). Taken together, these independent analyses show there are meaningful changes at the species level in bacteria in response to dietary FODMAP intake.

#### Metagenomic data analyses and genome assemblies revealed the Bifidobacterium niche expansion was specific for polyol-utilising strains

The sPLS-DA analysis of Pfam functional data revealed that the relative abundances of phosphotransferase systems predicted to be involved with polyol (sorbitol) utilisation were found to be discriminatory of the microbiota changes observed following consumption of the MFD, as well as *α* amylases and starch binding modules (online Supplementary Figure 3). The MGS data also enabled the recovery of 38 (LFD) and 46 (MFD) good to high-quality MAG (i.e., > 80 % completeness and < 10 % contamination, online Supplementary Table 3). The taxonomies represented within the MAG are consistent with the holistic analyses of the MGS datasets (via MetaPhlan2) and the 16S rRNA gene amplicon data (via the SILVA database). The genus *Bifidobacterium* produced the greatest number of MAG (5), with the two retrieved from the LFD datasets affiliated with *B. animalis,* and the three MAG from the MFD datasets representing *B. longum* and two strains of *B. adolescentis* (online Supplementary Figure 4). Furthermore, the carbohydrate-active enzyme profiles of these MAG validated the Pfam analysis of the MGS data, in that the *B. adolescentis* and *B. longum* MAG possess a greater gene count for polyol utilisation and sorbitol/mannitol metabolism than the *B. animalis* MAG recovered from the LFD group (online Supplementary Table 4). Taken together, these findings further validate and resolve that the composition of the MFD diet has redirected the Bifidobacteria populations towards those species favouring polyol metabolism for growth.

#### Correlation and network analyses revealed both intra- and inter–domain microbial interactions

**Figure 7.**
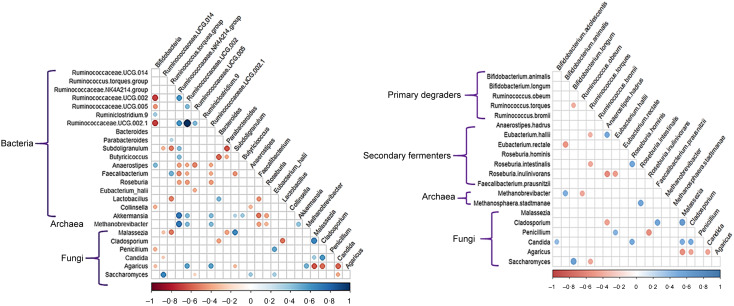
Spearman correlation analyses of the changes in relative abundance between the moderate FODMAP diet (MFD) and low FODMAP diet (LFD) (i.e. Δvalues) for the key bacterial, archaea and fungal taxa identified from (a) 16S rRNA and ITS2 profiles and (b) MGS and ITS2 profiles. Only those correlations with adjusted *P*-values < 0·05 following FDR correction are shown. Red circles denote negative correlations and blue circles denote positive correlations, with both colour intensity and the size of the circle proportional to the strength of the correlation.

Figure 7 shows the correlation matrices between ∆values of different fungal taxa (ITS2-based) with Bacterial/Archaeal taxa identified from the 16S rRNA gene amplicon or MGS datasets, respectively. The Δ*Bifidobacterium* values were positively correlated with Δ*Anaerostipes* and negatively correlated with Δ*Ruminococcaceae.* The Δ*Saccharomyces* values were positively correlated with the Δ*Anaerostipes* and Δ*E. hallii*, and the ΔArchaea *(Methanobrevibacter*) values were positively correlated with the Δ*Ruminococcaceae* and ∆*Akkermansia* values. In contrast, there was a strong negative correlation between the ∆ values for both *Methanobrevibacter* and *Akkermansia* (both hydrogen utilisers) with those for the genus *Faecalibacterium* and *Roseburia* (both butyrate producers). An even greater species-level resolution was observed using the MGS data (Figure 7(b)). There were statistically significant, positive correlations – suggesting co-associations – between the ∆ values of *Anaerostipes hadrus* and *E. hallii.* Positive fungus-bacteria correlations (co-associations) were found between the Δ values for *Saccharomyces* and *B. longum*, between *Candida* and *B. adolescentis* and between *Candida* and Δ*Roseburia hominis.* Positive Bacteria–Archaea correlations (co-associations) were found between the ∆ values for *Methanobrevibacter* and *B. animalis* and between *Methanosphaera stadtmanae* and *R. intestinalis.* Δ*R. hominis* and the archaeal species. Negative correlations – indicative of co-exclusions – were identified between the Δ values of *Saccharomyces* and *Ruminococcus torques,* as well as between *R.torques* and *B. longum*. Negative correlations (co-exclusions) between the Δ values of *B. animalis* and *E. rectale*, and between *Methanobrevibacter* and *Ruminococcus obeum*, were also observed. Taken together, the correlations based on the bacterial MGS data substantiate that species-level interrelationships can be obscured when using 16S rRNA amplicon data.

## Discussion

The specific effects of varying FODMAP content on the gastrointestinal physiology, symptom profile, psychology and microbiome of healthy people are not well documented beyond its effects on breath hydrogen and gastrointestinal symptoms^(33,65)^ and its modulation of some aspects of the stool microbiota via either dietary or individual FODMAP^(5)^. Here, the two diets that differed principally in their FODMAP content did not differ in the participant-reported outcomes of symptoms or scores on behavioural testing, or the characteristics of their stools in form, frequency or biochemical contents. However, gastrointestinal transit was faster with moderate compared with low FODMAP content. Deep analysis of the faecal bacterial and fungal communities in a comprehensive (inter-Kingdom) assessment of diet × microbiota interactions showed striking and contrasting effects on the species richness of the faecal bacterial and fungal communities.

Issues associated with study design require discussion. First, we chose the doses of FODMAP based upon the background reasons for the study – comparing ‘real-world’ strategies being observed in the community of restricting FODMAP to minimise gut symptoms to feel ‘healthier’ compared with increasing prebiotic intake that putatively might have health benefits. Marked increases in FODMAP intake were avoided given that such a strategy may induce symptoms even in healthy people. Second, we chose to perform a feeding study, in which meals were professionally prepared largely from primary ingredients and adherence was carefully monitored, to ensure tight dietary control, as we have utilised in previous studies^(38,68)^. The alternative of using supplements may not be relevant to whole-food dietary strategies. Third, a cross-over design with sufficient washout and adaptation time before outcomes associated with the new diet were measured was applied to minimise confounding from the considerable heterogeneity of gastrointestinal physiology and particularly gut microbiota across individuals. By limiting confounding factors, a sample size that was feasible (given the challenges associated with feeding studies^(69)^) and can generate meaningful results was achieved.

The dietary design and delivery were considered in general to be successful. Adherence was excellent and was confirmed objectively, at least in a sub-group of participants, by marked differences in breath hydrogen and methane generated, as previously well documented to occur with differing FODMAP intakes^(33)^. Differences in the diets were essentially restricted to the FODMAP content that spanned fructose in excess of glucose, fructans and GOS and the polyols, sorbitol and mannitol, where differences in intake between the diets differed four- to seven-fold. However, measurement of the content of what was actually eaten revealed a difference of about 5 g/d in the intake of dietary fibre (not including FODMAP). While this difference was not anticipated to influence the outcomes measured on the basis of previous studies in which fibre content was manipulated^(70,71)^, it must be considered in the interpretation of the findings.

The increase in FODMAP intake associated with MFD, while generating a larger amount of intestinal gas and presumably exerting a greater osmotic load on the small intestine, was not associated with the induction of gastrointestinal symptoms in this healthy cohort without gut complaints, as previously observed^(33,65)^. Previous reports of higher intake of FODMAP being associated with fatigue and depression were restricted to patients with IBS in a short-term controlled dietary intervention study^(33)^ or patients with lactose and/or fructose-induced abdominal symptoms^(34,35)^. The current study, however, supported the previous observation^(33)^ that, at least in the short term, differences in FODMAP intake do not impact fatigue or mood symptoms in healthy individuals.

There were no clinically discernible differences between the diets in their effects on stool frequency, volume and water content, which is not dissimilar to the lack of effects previously demonstrated with reduction of FODMAP intake in patients with IBS^(16)^. Similarly, faecal concentrations of SCFA, which derive from carbohydrate fermentation, and those of BCFA and phenols, which derive from protein fermentation, together with the luminal pH profile, were similar in association with each diet. While these faecal findings may appear paradoxical, the increased fermentation shown by the higher breath hydrogen concentrations is predominantly occurring in the proximal colon due to the ready fermentability of FODMAP. Greater distal colonic fermentative activity would not be anticipated as this largely reflects the polysaccharide fibre content of the diets and these only differed by about 5 g/d, which would not be expected to influence the results^(70,71)^. In most studies, faecal SCFA is not affected by alterations in dietary FODMAP content^(30)^ except where the fibre intake of the participants was not documented^(32)^.

Colonic transit was faster overall during the MFD, but this averaged at only a 10 % reduction in colonic transit time, a difference that would not be anticipated to affect substrate delivery or efficiency of absorption of metabolites. Previous studies have shown a spectrum of effects of individual FODMAP on gastric motility and emptying^(72,73)^. In those studies, however, the effects were not studied in the context of whole food where a spectrum of FODMAP is present. Hence, the observations in this study represent the sum total of these influences. Faster colonic transit in the MFD may relate to the increased water delivery to the colon^(74)^ and/or the increased carbohydrate fermentation that would deliver more SCFA to the proximal colon^(75,76)^. On the contrary, FODMAP-stimulated increase in methane production, as found in the minority who were methane producers, potentially slows rather than hastens colonic transit via its gasotransmitter actions^(77)^. While such an association was not evident in this study, the numbers of methane-producers was too small to reach any conclusions. The role of the modest increase in dietary fibre in the MFD may have influenced colonic transit times, but the lack of effect on faecal output and previous experience with fibre supplementation, albeit in patients with IBS, would not support measurable effects on transit in the colon^(70,71)^.

The effect of lowering FODMAP intake on the gut microbiota has been a lingering concern in the literature due to the reduction of dietary substrates with prebiotic actions^(5)^. In a meta-analysis of the effects of FODMAP on faecal microbiota, only *Bifidobacterium* spp. showed a consistent, statistically significantly greater abundance compared with that associated with a low FODMAP diet but no changes in Shannon *α* diversity in response to higher FODMAP intake^(30)^. These studies were generally impacted by the heterogeneity of the faecal microbiota across individuals and differences between simple supplementation vis-à-vis changes to a whole diet. In this study, these difficulties were mitigated by each subject acting as his/her own control and by the precision associated with dietary intake in each arm, discussed earlier. Our collective results suggest that the polyol content of the diet is rate limiting to the growth of those members of the Bifidobacteria that specialise in polyol utilisation. Indeed, there was an enrichment of polyol transport and utilisation genes in the MGS datasets from subjects consuming the MFD, and furthermore, MAG of polyol-utilising species of Bifidobacteria (*B. adolescentis* and *B. longum)* were recovered from the MFD datasets, whereas the *B. animalis* MAG recovered from the LFD datasets lacks these genes. The coordinate and increased relative abundances of *Eubacterium* and *Anaerostipes* with the MFD are most likely explained by their utilisation of *Bifidobacterium*-derived fermentation products such as lactate and acetate as well as other FODMAP degradation products, as ‘secondary fermenters’^(78–81)^.

In contrast to the bacteria, the fungal species count (richness) increased with the consumption of the MFD. While the increased signal for *Agaricus* is most likely of dietary origin, the genus *Saccharomyces* spp. were the most dominant and prevalent with both LFD and MFD, while *Candida –* reported to be positively associated with consumption of carbohydrate-rich diets by healthy humans^(79)^ *–* and *Aspergillus* spp. were only detectable when the MFD diet was consumed. As such, a key finding from our studies is that FODMAP oligosaccharides and/or polyols impact all Domains of microbial life inherent to the gut microbiome and need to be considered in the context of gut function and symptoms. To that end, our correlation analyses highlight positive relationships between *Saccharomyces* and the change in the abundance of *B. longum* and *Anaerostipes* spp, and *E. hallii.* We decided to retain within the datasets those reads assigned to the genus *Agaricus* to explore how the presence of this fungal biomass might affect the stool microbiota, and interestingly, some positive associations with presumptive specialist polysaccharide degraders such as *Ruminococcaceae*, and with methane-producer *Methanobrevibacter,* suggesting *Agaricus* may selectively promote fibre degraders and fermentations favoring methane formation. Other interrelationships between different fungal genera, and the between relative abundances of fungi and bacteria have been previously reported in the background of inflammatory bowel disease^(58)^ and in mice treated with antibiotics^(82)^ but not in the context of dietary components. Given the expanding interest in diet as a trigger of therapy for digestive and metabolic disease, these inter-domain (fungal × bacterial) interactions in response to FODMAP warrant greater attention.

The present study has the strengths of being carefully controlled in terms of the actual dietary intake, minimising confounders by its cross-over design, using robust methodologies and making observations that were, in general, statistically powerful. The study has weaknesses. First, the translation of the findings to the real-world setting where diets are often not of good quality and do not meet the healthy eating guidelines, or to patients with IBS, is uncertain, limiting the generalisability of the findings. Second, greater fibre content of the MFD may have confounded some of the findings. Third, there is a possibility of carry-over effects from the cross-over design, though none were observed. Also, true counterbalancing is not possible with an odd number of participants. Fourth, the short-term nature of the dietary interventions does not permit longer term effects of strict FODMAP restrictions on gut or psychological health to be addressed. Fifth, the number of participants was relatively small even though many confounders were minimised by the cross-over design. Sample size was challenging to estimate given the lack of studies in the healthy population. The numbers studied were also limited by the failure to adequately collect faeces in seven participants and the technological issues associated with the wireless motility capsule. Hence, the interpretation of the lack of effects for many endpoints must be guarded for this reason.

The implications of the current findings are that, within the time frame of this study, a modest increase (or decrease) in the daily intake of dietary FODMAP in healthy adults does not result in noticeable (and quantifiable) changes in their gut function or mental health outcomes. In contrast, there were measurable changes in the compositional attributes of the fungal and bacterial communities of these subjects in response to dietary FODMAP content and emphasises the need to quantitatively assess all microbial domains present within the ‘gut microbiome’ and their interrelationships. Importantly though, and within the time frame of this study, such changes to the microbiome did not translate into significant alterations in the fecal indices measured here and deemed relevant to assessing gut homeostasis, inflammation and health. As such, our results show that while changes to microbiome composition can be relatively rapid in response to dietary FODMAP intake, the time between these changes and their measurable impact on faecal biomarkers (and measures of gut health) require considerably longer observation periods than a few weeks. Hence, future work should include an examination of how long it takes a sustained change to the gut microbiome in response to dietary FODMAP intake to ultimately effect measurable changes to fecal indices of gut health, and in turn, how rapidly it reverts after cessation of that level of intake.

In conclusion, this study has reinforced the resilience and adaptability of the healthy adults not to manifest alterations in gastrointestinal symptoms and stool characteristics despite modest changes in regional gastrointestinal transit and considerable alteration in the microbial community. Our findings show that high food-associated polyol intake is rate-limiting to the growth of key members of the genus, such as *B. longum* and *B. adolescentis,* which provides strategies to either augment this population by using specific probiotic *Bifidobacterium* strains adapted to diets with a low FODMAP content, or by dietary liberalisation to provide a small daily intake of select polyols. This study also shows that the FODMAP content of the diet affects the gut mycobiome in healthy individuals. The taxonomy-based shifts were reflected in lower bacterial, but increased fungal, richness in response to the MFD. These inter-domain relationships are relevant to improving our understanding of the consequences of diet on gut function. Hence, within the limitation of the outcomes measured, short-term alteration to dietary FODMAP intake does not influence healthy adults to feel healthier, but the unknown consequences on gut function of the differences in the gut microbiota – both bacteria and fungal – over the longer term deserve further attention.

## Supporting information

Murtaza et al. supplementary material Murtaza et al. supplementary material

## Supplementary material

For supplementary material/s referred to in this article, please visit https://doi.org/10.1017/S0007114525000868

## References

[1] Lee AR (2022) Review article: dietary management of coeliac disease. Aliment Pharmacol Ther 56, S38–S48. 35815831 10.1111/apt.16974

[2] Biesiekierski JR , Muir JG & Gibson PR (2013) Is gluten a cause of gastrointestinal symptoms in people without celiac disease? Curr Allergy Asthma Rep 13, 631–638. 24026574 10.1007/s11882-013-0386-4

[3] Aziz I , Dwivedi K & Sanders DS (2016) From coeliac disease to noncoeliac gluten sensitivity; should everyone be gluten free? Curr Opin Gastroenterol 32, 120–127. 26808363 10.1097/MOG.0000000000000248

[4] Sperber AD , Bangdiwala SI , Drossman DA , et al. (2021) Worldwide prevalence and burden of functional gastrointestinal disorders, results of Rome Foundation Global Study. Gastroenterology 160, 99–114. 32294476 10.1053/j.gastro.2020.04.014

[5] Gibson PR , Halmos EP & Muir JG (2020) Review article: FODMAPS, prebiotics and gut health-the FODMAP hypothesis revisited. Aliment Pharmacol Ther 52, 233–246. 32562590 10.1111/apt.15818

[6] Black CJ , Staudacher HM & Ford AC (2022) Efficacy of a low FODMAP diet in irritable bowel syndrome: systematic review and network meta-analysis. Gut 71, 1117–1126. 34376515 10.1136/gutjnl-2021-325214

[7] Moayyedi P , Andrews CN , MacQueen G , et al. (2019) Canadian Association of Gastroenterology clinical practice guideline for the management of Irritable Bowel Syndrome (IBS). J Can Assoc Gastroenterol 2, 6–29. 31294724 10.1093/jcag/gwy071 PMC6507291

[8] Chey WD , Hashash JG , Manning L , et al. (2022) AGA clinical practice update on the role of diet in irritable bowel syndrome: expert review. Gastroenterology 162, 1737–1745. 35337654 10.1053/j.gastro.2021.12.248

[9] Palsson OS , Tack J , Drossman DA , Le Nevé B , et al. (2024) Worldwide population prevalence and impact of sub-diagnostic gastrointestinal symptoms. Aliment Pharmacol Ther 59, 852–864. 38311841 10.1111/apt.17894

[10] Gibson PR , Halmos EP , So D , et al. (2022) Diet as a therapeutic tool in chronic gastrointestinal disorders: lessons from the FODMAP journey. J Gastroenterol Hepatol 37, 644–652. 34994019 10.1111/jgh.15772

[11] Hyams JS & Leichtner AM (1985) Apple juice. An unappreciated cause of chronic diarrhea. Am J Dis Child 139, 503–505. 3984976 10.1001/archpedi.1985.02140070077039

[12] Schumann D , Klose P , Lauche R , et al. (2018) Low fermentable, oligo-, di-, mono-saccharides and polyol diet in the treatment of irritable bowel syndrome: a systematic review and meta-analysis. Nutrition 45, 24–31. 29129233 10.1016/j.nut.2017.07.004

[13] Goyal O , Batta S , Nohria S , et al. (2021) Low fermentable oligosaccharide, disaccharide, monosaccharide, and polyol diet in patients with diarrhea-predominant irritable bowel syndrome: a prospective, randomized trial. J Gastroenterol Hepatol 36, 2107–2115. 33464683 10.1111/jgh.15410

[14] Bohn L , Storsrud S , Liljebo T , et al. (2015) Diet low in FODMAPs reduces symptoms of irritable bowel syndrome as well as traditional dietary advice: a randomized controlled trial. Gastroenterology 149, 1399–1407. 26255043 10.1053/j.gastro.2015.07.054

[15] Pedersen N , Andersen NN , Vegh Z , et al. (2014) Ehealth: low FODMAP diet *v.* lactobacillus rhamnosus GG in irritable bowel syndrome. World J Gastroenterol 20, 6215–16226. 10.3748/wjg.v20.i43.16215 PMC4239510 25473176

[16] Halmos EP , Biesiekierski JR , Newnham ED , et al. (2018) Inaccuracy of patient-reported descriptions of and satisfaction with bowel actions in irritable bowel syndrome. Neurogastroenterol Motil 30, e13187. 10.1111/nmo.13187 28799291

[17] Dean G , Chey SW , Singh P , et al. (2024) A diet low in fermentable oligo-, di-, monosaccharides and polyols improves abdominal and overall symptoms in persons with all subtypes of irritable bowel syndrome. Neurogastroenterol Motil 36, e14845. 38887150 10.1111/nmo.14845

[18] Bovee-Oudenhoven IM , ten Bruggencate SJ , Lettink-Wissink ML , et al. (2003) Dietary fructo-oligosaccharides and lactulose inhibit intestinal colonisation but stimulate translocation of Salmonella in rats. Gut 52, 1572–1578. 14570725 10.1136/gut.52.11.1572 PMC1773861

[19] Petersen A , Heegaard PMH , Pedersen AL , et al. (2009) Some putative prebiotics increase the severity of Salmonella enterica serovar Typhimurium infection in mice. BMC Microbiol 9, 245. 19948011 10.1186/1471-2180-9-245 PMC2789089

[20] Kamphuis JBJ , Guiard B , Leveque M , et al. (2020) Lactose and fructo-oligosaccharides increase visceral sensitivity in mice via glycation processes, increasing mast cell density in colonic mucosa. Gastroenterology 158, 652–663. 31711923 10.1053/j.gastro.2019.10.037

[21] Ten Bruggencate SJ , Bovee-Oudenhoven IM , Lettink-Wissink ML , et al. (2003) Dietary fructo-oligosaccharides dose-dependently increase translocation of salmonella in rats. J Nutr 133, 2313–2318. 12840199 10.1093/jn/133.7.2313

[22] Ten Bruggencate SJ , Bovee-Oudenhoven IM , Lettink-Wissink ML , et al. (2004) Dietary fructo-oligosaccharides and inulin decrease resistance of rats to Salmonella: protective role of calcium. Gut 53, 530–535. 15016747 10.1136/gut.2003.023499 PMC1774012

[23] Zhou S-Y , Gillilland M , Wu X , et al. (2018) FODMAP diet modulates visceral nociception by lipopolysaccharide-mediated intestinal inflammation and barrier dysfunction. J Clin Invest 128, 267–280. 29202473 10.1172/JCI92390 PMC5749529

[24] Singh P , Grabauskas G , Zhou SY , et al. (2021) High FODMAP diet causes barrier loss via lipopolysaccharide-mediated mast cell activation. JCI Insight 6, e146529. 34618688 10.1172/jci.insight.146529 PMC8663790

[25] Hustoft TN , Hausken T , Ystad SO , et al. (2017) Effects of varying dietary content of fermentable short-chain carbohydrates on symptoms, fecal microenvironment, and cytokine profiles in patients with irritable bowel syndrome. Neurogastroenterol Motil 29, e12969. 10.1111/nmo.12969 27747984

[26] Ten Bruggencate SJM , Bovee-Oudenhoven IMJ , Lettink-Wissink MLG , et al. (2006) Dietary fructooligosaccharides affect intestinal barrier function in healthy men. J Nutr 136, 70–74. 16365061 10.1093/jn/136.1.70

[27] Swanson KS , Gibson GR , Hutkins R , et al. (2020) The International Scientific Association for Probiotics and Prebiotics (ISAPP) consensus statement on the definition and scope of synbiotics. Nat Rev Gastroenterol Hepatol 17, 687–701. 32826966 10.1038/s41575-020-0344-2 PMC7581511

[28] Slomski A (2020) The low-FODMAP diet helps IBS symptoms, but questions remain. JAMA 323, 1029–1031. 32101250 10.1001/jama.2020.0691

[29] Bellini M & Rossi A (2018) Is a low FODMAP diet dangerous? Tech Coloproctol 22, 569–571. 30083779 10.1007/s10151-018-1835-9

[30] So D , Loughman A & Staudacher HM (2022) Effects of a low FODMAP diet on the colonic microbiome in irritable bowel syndrome: a systematic review with meta-analysis. Am J Clin Nutr 116, 943–952. 35728042 10.1093/ajcn/nqac176 PMC9535515

[31] Vervier K , Moss S , Kumar N , et al. (2022) Two microbiota subtypes identified in irritable bowel syndrome with distinct responses to the low FODMAP diet. Gut 71, 1821–1830. 34810234 10.1136/gutjnl-2021-325177 PMC9380505

[32] Valeur J , Røseth AG , Knudsen T , et al. (2016) Fecal fermentation in irritable bowel syndrome: influence of dietary restriction of fermentable oligosaccharides, disaccharides, monosaccharides and polyols. Digestion 94, 50–56. 27487397 10.1159/000448280

[33] Ong DK , Mitchell SB , Barrett JS , et al. (2010) Manipulation of dietary short chain carbohydrates alters the pattern of gas production and genesis of symptoms in irritable bowel syndrome. J Gastroenterol Hepatol 25, 1366–1373. 20659225 10.1111/j.1440-1746.2010.06370.x

[34] Ledochowski M , Sperner-Unterweger B & Fuchs D (1998) Lactose malabsorption is associated with early signs of mental depression in females: a preliminary report. Dig Dis Sci 43, 2513–2517. 9824144 10.1023/a:1026654820461

[35] Ledochowski M , Sperner-Unterweger B , Widner B , et al. (1998) Fructose malabsorption is associated with early signs of mental depression. Eur J Med Res 3, 295–298. 9620891

[36] Zhang Q , Chen B , Zhang J , et al. (2023) Effect of prebiotics, probiotics, synbiotics on depression: results from a meta-analysis. BMC Psychiatry 23, 477. 37386630 10.1186/s12888-023-04963-x PMC10308754

[37] Dwan K , Li T , Altman DG , et al. (2019) CONSORT 2010 statement: extension to randomised crossover trials. Br Med J 366, l4378. 31366597 10.1136/bmj.l4378 PMC6667942

[38] Halmos EP , Power VA , Shepherd SJ , et al. (2014) A diet low in FODMAPs reduces symptoms of irritable bowel syndrome. Gastroenterology 146, 67–75. 24076059 10.1053/j.gastro.2013.09.046

[39] Varney J , Barrett J , Scarlata K , et al. (2017) FODMAPs: food composition, defining cutoff values and international application. J Gastroenterol Hepatol 32, 53–61. 10.1111/jgh.13698 28244665

[40] Barrett JS & Gibson PR (2010) Development and validation of a comprehensive semi-quantitative food frequency questionnaire that includes FODMAP intake and glycemic index. J Am Diet Assoc 110, 1469–1476. 20869485 10.1016/j.jada.2010.07.011

[41] National Health and Medical Research Council (2013) Australian Dietary Guidelines. https://www.health.gov.au/resources/publications/the-australian-dietary-guidelines?language=en (accessed June 2015).

[42] Biesiekierski JR , Rosella O , Rose R , et al. (2011) Quantification of fructans, galacto-oligosacharides and other short-chain carbohydrates in processed grains and cereals. J Hum Nutr Diet 24, 154–176. 21332832 10.1111/j.1365-277X.2010.01139.x

[43] Muir JG , Rose R , Rosella O , et al. (2009) Measurement of short-chain carbohydrates in common Australian vegetables and fruits by high-performance liquid chromatography (HPLC). J Agric Food Chem 57, 554–565. 19123815 10.1021/jf802700e

[44] Tuck C , Ly E , Bogatyrev A , et al. (2018) Fermentable short chain carbohydrate (FODMAP) content of common plant-based foods and processed foods suitable for vegetarian- and vegan-based eating patterns. J Hum Nutr Diet 31, 422–435. 29473657 10.1111/jhn.12546

[45] Landon S , Colyer CGB & Salman H (2012) *The Resistant Starch Report*. North Sydney, Australia: Australian Institute of Food Science and Technology.

[46] Monteiro CA , Cannon G , Moubarac JC , et al. (2018) The UN Decade of Nutrition, the NOVA food classification and the trouble with ultra-processing. Public Health Nutr 21, 5–17. 28322183 10.1017/S1368980017000234 PMC10261019

[47] Saad RJ & Hasler WL (2011) A technical review and clinical assessment of the wireless motility capsule. Gastroenterol Hepatol (NY) 7, 795–804. PMC3280411 22347818

[48] Arora Z , Parungao JM , Lopez R , et al. (2015) Clinical utility of wireless motility capsule in patients with suspected multiregional gastrointestinal dysmotility. Dig Dis Sci 60, 1350–1357. 25399332 10.1007/s10620-014-3431-9

[49] Thwaites PA , Yao CK , Maggo J , et al. (2022) Comparison of gastrointestinal landmarks using the gas-sensing capsule and wireless motility capsule. Aliment Pharmacol Ther 56, 1337–1348. 36082475 10.1111/apt.17216 PMC9826325

[50] Spielberger C (1995) State-Trait Personality Inventory (STPI) Research Manual Sampler Set. Menlo Park: Mind Garden Inc.

[51] Lovibond, SH & Lovibond PF (1995) Manual for the Depression Anxiety & Stress Scales, 2nd ed. Sydney: Psychology Foundation.

[52] Fisk JD & Doble SE (2002) Construction and validation of a fatigue impact scale for daily administration (D-FIS). Qual Life Res 11, 263–272. 12074263 10.1023/a:1015295106602

[53] Gill PA , Muir JG , Gibson PR , et al. (2022) A randomized dietary intervention to increase colonic and peripheral blood SCFAs modulates the blood B- and T-cell compartments in healthy humans. Am J Clin Nutr 116, 1354–1367. 36084000 10.1093/ajcn/nqac246 PMC9630882

[54] Yoshikawa M , Taguchi Y , Arashidani K , et al. (1986) Determination of cresols in urine by high-performance liquid chromatography. J Chromatogr 362, 425–429. 3760050 10.1016/s0021-9673(01)86996-x

[55] Yu Z & Morrison M (2004) Improved extraction of PCR-quality community DNA from digesta and fecal samples. Biotechniques 36, 808–812. 15152600 10.2144/04365ST04

[56] Shanahan ER , Shah A , Koloski N , et al. (2018) Influence of cigarette smoking on the human duodenal mucosa-associated microbiota. Microbiome 6, 150. 30157953 10.1186/s40168-018-0531-3 PMC6116507

[57] Murtaza N , Burke LM , Vlahovich N , et al. (2019) The effects of dietary pattern during intensified training on stool microbiota of elite race walkers. Nutrients 11, 261. 30682843 10.3390/nu11020261 PMC6413084

[58] Sokol H , Leducq V , Aschard H , et al. (2017) Fungal microbiota dysbiosis in IBD. Gut 66, 1039–1048. 26843508 10.1136/gutjnl-2015-310746 PMC5532459

[59] Quast C , Pruesse E , Yilmaz P , et al. (2013) The SILVA ribosomal RNA gene database project: improved data processing and web-based tools. Nucleic Acids Res 41, D590–D596. 23193283 10.1093/nar/gks1219 PMC3531112

[60] Nilsson RH , Larsson KH , Taylor AFS , et al. (2019) The UNITE database for molecular identification of fungi: handling dark taxa and parallel taxonomic classifications. Nucleic Acids Res 47, D259–D264. 30371820 10.1093/nar/gky1022 PMC6324048

[61] Franzosa EA , McIver LJ , Rahnavard G , et al. (2018) Species-level functional profiling of metagenomes and metatranscriptomes. Nat Methods 15, 962–968. 30377376 10.1038/s41592-018-0176-y PMC6235447

[62] Truong DT , Franzosa EA , Tickle TL , et al. (2015) MetaPhlAn2 for enhanced metagenomic taxonomic profiling. Nat Methods 12, 902–903. 26418763 10.1038/nmeth.3589

[63] Wattam AR , Davis JJ , Assaf R , et al. (2017) Improvements to PATRIC, the all-bacterial Bioinformatics Database and Analysis Resource Center. Nucleic Acids Res 45, D535–D542. 27899627 10.1093/nar/gkw1017 PMC5210524

[64] Ondov BD , Treangen TJ , Melsted P , et al. (2016) Mash: fast genome and metagenome distance estimation using MinHash. Genome Biol 17, 132. 27323842 10.1186/s13059-016-0997-x PMC4915045

[65] Halmos EP , Christophersen CT , Bird AR , et al. (2015) Diets that differ in their FODMAP content alter the colonic luminal microenvironment. Gut 64, 93–100. 25016597 10.1136/gutjnl-2014-307264

[66] Zakrzewski M , Proietti C , Ellis JJ , et al. (2017) Calypso: a user-friendly web-server for mining and visualizing microbiome–environment interactions. Bioinf 33, 782–783. 10.1093/bioinformatics/btw725 PMC5408814 28025202

[67] Lê Cao KA , Boitard S & Besse P (2011) Sparse PLS discriminant analysis: biologically relevant feature selection and graphical displays for multiclass problems. BMC Bioinf 12, 253. 10.1186/1471-2105-12-253 PMC3133555 21693065

[68] Biesiekierski JR , Peters SL , Newnham ED , et al. (2013) No effects of gluten in patients with self-reported non-celiac gluten sensitivity after dietary reduction of fermentable, poorly absorbed, short-chain carbohydrates. Gastroenterology 145, 320–328. 23648697 10.1053/j.gastro.2013.04.051

[69] Yao CK , Gibson PR & Shepherd SJ (2013) Design of clinical trials evaluating dietary interventions in patients with functional gastrointestinal disorders. Am J Gastroenterol 108, 748–758. 23609614 10.1038/ajg.2013.77

[70] So D , Yao CK , Ardalan ZS , et al. (2022) Supplementing dietary fibers with a low FODMAP diet in irritable bowel syndrome: a randomized controlled crossover trial. Clin Gastroenterol Hepatol 20, 2112–2120. 34929392 10.1016/j.cgh.2021.12.016

[71] So D , Yao CK , Gill PA , et al. (2023) Detection of changes in regional colonic fermentation in response to supplementing a low FODMAP diet with dietary fibres by hydrogen concentrations, but not by luminal pH. Aliment Pharmacol Ther 58, 417–428. 37386938 10.1111/apt.17629 PMC10946934

[72] Clegg M & Shafat A (2010) Gastric emptying and orocaecal transit time of meals containing lactulose or inulin in men. Br J Nutr 104, 554–559. 20370945 10.1017/S0007114510000905

[73] Masuy I , Van Oudenhove L , Tack J , et al. (2018) Effect of intragastric FODMAP infusion on upper gastrointestinal motility, gastrointestinal, and psychological symptoms in irritable bowel syndrome *v.* healthy controls. Neurogastroenterol Motil 30, e13167. 10.1111/nmo.13167 28762592

[74] Spiller R (2017) How do FODMAPs work? J Gastroenterol Hepatol 32, 36–39. 28244663 10.1111/jgh.13694

[75] Cherbut C , Aubé AC , Blottière HM , et al. (1997) Effects of short-chain fatty acids on gastrointestinal motility. Scand J Gastroenterol Suppl 222, 58–61. 9145449 10.1080/00365521.1997.11720720

[76] Fukumoto S , Tatewaki M , Yamada T , et al. (2003) Short-chain fatty acids stimulate colonic transit via intraluminal 5-HT release in rats. Am J Physiol Regul Integr Comp Physiol 284, R1269–R1276. 12676748 10.1152/ajpregu.00442.2002

[77] Triantafyllou K , Chang C & Pimentel MZ (2014) Methanogens, methane and gastrointestinal motility. J Neurogastroenterol Motil 20, 31–40. 24466443 10.5056/jnm.2014.20.1.31 PMC3895606

[78] Rivière A , Gagnon M , Weckx S , et al. (2015) Mutual cross–feeding interactions between Bifidobacterium longum subsp. longum NCC2705 and Eubacterium rectale ATCC 33656 explain the bifidogenic and butyrogenic effects of arabinoxylan oligosaccharides. Appl Environ Microbiol 81, 7767–7781. 26319874 10.1128/AEM.02089-15 PMC4616955

[79] Scardovi V (1965) The fructose-6-phosphate shunt as a peculiar pattern of hexose degradation in the genus Bifidobacterium. Ann Microbiol Enzym 15, 19–29.

[80] Duncan SH , Louis P & Flint HJ (2004) Lactate-utilizing bacteria, isolated from human feces, that produce butyrate as a major fermentation product. Appl Environ Microbiol 70, 5810–5817. 15466518 10.1128/AEM.70.10.5810-5817.2004 PMC522113

[81] Hoffmann C , Dollive S , Grunberg S , et al. (2013) Archaea and fungi of the human gut microbiome: correlations with diet and bacterial residents. PLoS One 8, e66019. 23799070 10.1371/journal.pone.0066019 PMC3684604

[82] Dollive S , Chen YY , Grunberg S , et al. (2013) Fungi of the murine gut: episodic variation and proliferation during antibiotic treatment. PLoS One 8, e71806. 23977147 10.1371/journal.pone.0071806 PMC3747063

