# Supplementary material for: Effects of dietary FODMAP content on the faecal microbiome and gastrointestinal physiology in healthy adults: a randomised, controlled cross-over feeding study
Source: Br J Nutr. 2025 Apr 24;134(9):712–26. doi: 10.1017/S0007114525000868 (PMC12766111; doi:10.1017/S0007114525000868)
Supplement: Murtaza et al. supplementary material [file S0007114525000868sup001.docx]

**Effects of dietary FODMAP content on the faecal microbiome and gastrointestinal physiology in healthy adults: a randomised, controlled cross-over feeding study**

**Nida Murtaza**^2^**, Lyndal Collins**^1^**, Chu K. Yao**^1^**, Phoebe A. Thwaites**^1^**, Patricia Veitch**^1^**, Jane E. Varney**^1^**, Paul A. Gill**^1^**, Peter R Gibson**^1*^**, Mark Morrison**^2*^**, Jane G. Muir**^1*^

**SUPPLEMENTARY MATERIAL**

**Supplementary methodological information:**

- Details of questionnaires used for assessment of mental health and fatigue
- Analysis of faecal microbiota

**Supplementary Figures 1-4**

**Supplementary Tables 1-4**

**QUESTIONNAIRES FOR ASSESSMENT OF MENTAL HEALTH AND FATIGUE**

During the last week of each dietary arm, participants completed three questionnaires:

- *State-Trait-Personality Inventory (STPI)*^(1)^: This is an 80-item self-report questionnaire, with eight 10-item scales for measuring state and trait anxiety, depression, anger and curiosity. State items are used to assess current emotional state and are rated on a four-point intensity scale, where 1=not at all; and 4= very much so. Trait items assess emotional disposition and are rated on a four-point intensity scale, where 1= almost never; and 4= almost always. The range of possible scores for each subscale can vary from a minimum of 10 to a maximum of 40.
- *Abbreviated Depression Anxiety Severity Scale (DASS-21)*^(2)^]: This validated abbreviated version of the DASS, a set of scales designed to assess depression, anxiety and stress, comprised of 21 items. Responses are recorded via a four-point severity scale, with total scores for each domain derived by summing the responses for their respective items. Higher scores represent greater severity; the maximum possible score for each domain is 21.
- *Daily Fatigue Impact Scale (D-FIS)*^(3)^:This asks the subject to rate how much of a problem fatigue has caused them in the past month in reference to each item’s statement by circling one of the following responses: 0=no problem, 1=small problem, 2=moderate problem, 3=big problem, 4=extreme problem. The 40-item scale encompasses physical (10 items), cognitive (10 items) and psychosocial domains (20 items). A total score is generated by summing the ordinal ratings, where higher scores represent greater impact of fatigue on cognition, physical functioning and daily activities; the maximum possible score is 32

**REFERENCES**

1. Spielberger C (1995) *State-Trait Personality Inventory (STPI) research manual sampler set*. Menlo Park: Mind Garden Inc.
2. Lovibond, SH. & Lovibond PF (1995) *Manual for the Depression Anxiety & Stress Scales*, 2^nd^ ed. Sydney: Psychology Foundation.
3. Fisk JD & Doble SE (2002) Construction and validation of a fatigue impact scale for daily administration (D-FIS). *Qual Life Res* **11**, 263-272.

**ANALYSIS OF FAECAL MICROBIOTA**

During stool collection, a subsample was transferred by the subject to OMNIgene GUT sample preservation tubes (OMNIgene, DNA Genotek, Ottawa, Canada) using their recommended protocol. These tubes were temporarily stored at 4 ^o^C before long term storage at -80 ^o^C. For DNA extraction, the tubes were allowed to equilibrate to room temperature, their contents mixed and then subsamples (~250 mg) was aseptically transferred to 2 ml screw-capped tubes and processed using the repeated bead-beating procedure^(1)^ adapted for use with the Precelleys tissue homogeniser (Bertin Technologies) and combined with an automated column-based DNA purification procedure (Maxwell® 16MDx system, Promega Corporation, WI, USA) as previously described^(2)^. The DNA was then used to produce amplicon libraries of the V6-V8 hypervariable regions from the gene encoding 16S rRNA, with the Bacteria/Archaea specific primers 926F and 1392R, according to the previously published protocols^(3)^. In parallel, a second DNA sample was used for PCR-based amplification of the intergenic transcribed spacer region 2 (ITS-2) using the forward and reverse primers described by Sokol et al.^(4)^. Both these amplicon libraries were further processed, bar-coded and pooled for Illumina MiSeq sequencing. A third subsample of the DNA was randomly sheared, end-repaired, and used to construct the libraries necessary for shotgun metagenomic sequencing (MGS) via NextSeq 500/550 High Output v2 (2 x 150 bp paired end) chemistry, and to provide 3 Gbp sequencing depth. All the DNA processing, library construction, and sequencing steps were performed by The University of Queensland’s Australian Centre of Ecogenomics (ACE, [www.ecogenomic.org](http://www.ecogenomic.org)) using their standard protocols.

**16S rRNA and ITS2 amplicon, and MGS bioinformatics workflows**

The raw sequence reads were first processed with FastQC (version 0.11.4, https://www.bioinformatics.babraham.ac.uk/projects/fastqc/), and then cutadapt (version 1.17) was used to remove those reads lacking primer sequences^(5)^. Low-quality reads were removed using Trimmomatic v0.32^(6)^ set with a sliding window of four bases using Q-score criterion. The remaining reads were then hard-trimmed to 250 nucleotides, and those below this threshold were also removed. The forward reads remaining following this procedure were then processed via QIIME2 for feature selection, abundance calculations, and taxonomy assignments. First, the reads were de-noised (i.e., filtered, dereplicated, and chimeras identified and removed) using DADA2 (--p-trunc-len = 0)^(3)^ and the relative frequencies of each resulting “representative feature sequence” were calculated.

The taxonomies for these representative feature sequences were assigned by BLASTn alignments using the classify-consensus-blast function with default parameters, with each sequence compared to either the non-redundant SILVA (for 16S rRNA gene profiling: release 132, clustered at 99% identity ^(7)^) or UNITE (for ITS2 profiling: version 7.2, clustered at 99% identity^(8)^) databases. This feature table was then filtered to remove any sequences with a relative abundance of less than 0.01%, as well as those also deemed present in the control samples (i.e. reagents only), to produce a “filtered feature” table. Samples with less than 1000 reads were also removed from further analysis, of which 4 samples were excluded from the ITS2 dataset. The filtered feature table was rarefied to 16878 reads for 16S rRNA and to 1745 reads for fungal ITS2 sequence analysis.

For the MGS data, the Illumina sequence adaptors were first trimmed from the raw sequences, and the forward and reverse reads concatenated into a single FASTQ file for each sample. Any reads of human origin were removed from the datasets using bowtie2 (version 2.2.1) relative to the human (hg19) database^(9)^. The remaining sequences were then subjected to taxonomic and functional pathway profiling using the HUMAnN2 pipeline^(10)^. Taxonomy profiles predicted from the MGS data were produced using MetaPhlAn2^(11)^, and functional pathways were assigned to the MGS data using ChocoPhlAn as the reference pangenome database and UniRef50 as the protein reference database (i.e., assignments made for those reads with >50% identity). The resultant table was exported to Calypso for downstream statistical analyses^(12)^. FastQC was used to check the quality and Nonpareil methodology was used to estimate the coverage of the metagenomic data^(13,14)^.

**Recovery and comparative analysis of metagenome-assembled genomes (MAG)**

The quality checked paired-end reads were assembled using MEGAHIT (version 1.1.1)^(15)^, and BamM (version 1.7.3) was used for read-mapping and the generation of contiguous sequences (contigs). MetaBAT (version 0.32.4) was then used for binning and to recover the MAG^(16)^. The quality of the recovered MAG in terms of their predicted completeness and contamination was assessed using CheckM (version 1.0.7)^(17)^, and those MAG with scores >80% for completeness and <10% contamination were selected for further analyses. The MAG were first uploaded to the PATRIC and their taxonomy was predicted using the Similar Genome Finder service, which computes genome distance estimation using the MinHash (Mash) algorithm based on hierarchical clustering to the public genomes that are available on PATRIC^(18,19)^. The Average Nucleotide Identity (ANI) matrix among the Bifidobacterium-affiliated MAG was constructed using FASTANI, and carbohydrate degrading enzymes for each MAG were predicted using PATRIC.

**REFERENCES**

1. Yu Z & Morrison M (2004) Improved extraction of PCR-quality community DNA from digesta and fecal samples. *Biotechniques* **36**, 808-812.
2. Shanahan ER, Shah A, Koloski N, et al. (2018) Influence of cigarette smoking on the human duodenal mucosa-associated microbiota. *Microbiome* **6**, 150.
3. Murtaza N, Burke LM, Vlahovich N, et al. (2019) The effects of dietary pattern during intensified training on stool microbiota of elite race walkers. *Nutrients* **11**, 261.
4. Sokol H, Leducq V, Aschard H, et al. (2017) Fungal microbiota dysbiosis in IBD. *Gut* **66**, 1039-1048.
5. Martin M (2011) Cutadapt removes adapter sequences from high-throughput sequencing reads. *EMBnet.journal* **17**, 10-12.
6. Bolger AM, Lohse M, Usadel B (2014) Trimmomatic: a flexible trimmer for Illumina sequence data. *Bioinformatics* **30**, 2114-2120.
7. Quast C, Pruesse E, Yilmaz P, et al. (2013) The SILVA ribosomal RNA gene database project: improved data processing and web-based tools. *Nucleic Acids Res* **41**, D590-D596.
8. Nilsson RH, Larsson KH, Taylor AFS, et al. (2019) The UNITE database for molecular identification of fungi: handling dark taxa and parallel taxonomic classifications. *Nucleic Acids Res* **47**, D259-D264.
9. Langmead B & Salzberg SL (2012) Fast gapped-read alignment with Bowtie 2. *Nat Methods* **9**, 357-359.
10. Franzosa EA, McIver LJ, Rahnavard G, et al. (2018) Species-level functional profiling of metagenomes and metatranscriptomes. *Nat Methods* **15**, 962-968.
11. Truong DT, Franzosa EA, Tickle TL, et al. (2015) MetaPhlAn2 for enhanced metagenomic taxonomic profiling. *Nat Methods* **12**, 902-903.
12. Zakrzewski M, Proietti C, Ellis JJ, et al. (2017) Calypso: a user-friendly web-server for mining and visualizing microbiome-environment interactions. *Bioinformatics* **33**, 782-783.
13. Babraham Bioinformatics. FastQC. A quality control tool for high throughput sequence data. <http://www.bioinformatics.babraham.ac.uk/projects/fastqc/> (accessed 26/9/2019).
14. Rodriguez-R LM, Gunturu S, Tiedje JM, et al. (2018) Nonpareil 3: fast estimation of metagenomic coverage and sequence diversity. *mSystems* **3**, e00039-18.
15. Li D, Liu CM, Luo R, et al. (2015) MEGAHIT: an ultra-fast single-node solution for large and complex metagenomics assembly via succinct de Bruijn graph. *Bioinformatics* **31**, 1674-1676.
16. Kang DD, Froula J, Egan R, et al. (2015) MetaBAT, an efficient tool for accurately reconstructing single genomes from complex microbial communities. *PeerJ* **3**, e1165.
17. Parks DH, Imelfort M, Skennerton CT, et al. (2015) CheckM: assessing the quality of microbial genomes recovered from isolates, single cells, and metagenomes. *Genome Res* **25**, 1043-1055.
18. Wattam AR, Davis JJ, Assaf R, et al. (2017) Improvements to PATRIC, the all-bacterial Bioinformatics Database and Analysis Resource Center. *Nucleic Acids Res* **45**, D535-D542.
19. Ondov BD, Treangen TJ, Melsted P, et al. (2016) Mash: fast genome and metagenome distance estimation using MinHash. *Genome Biol* **17**, 132.

**SUPPLEMENTARY FIGURES 1-4**

**Supplementary Figure 1.** Areas under the curve for hydrogen and methane of hourly breath tests for 12 hours during the low FODMAP diet (LFD) and moderate FODMAP diet (MFD).


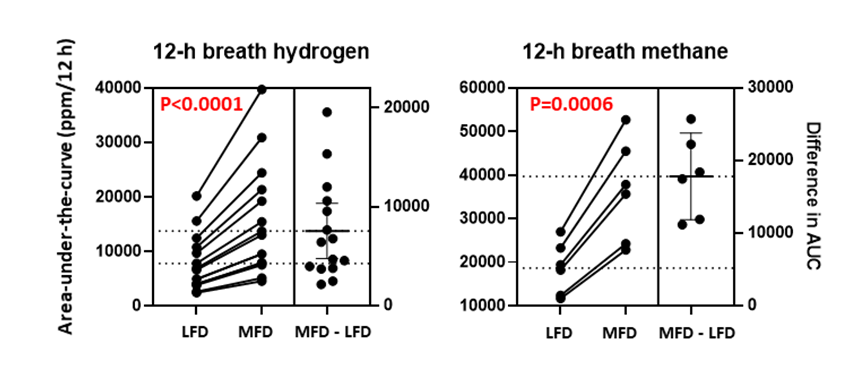


**Supplementary Figure 2.** Differences in the genera in faeces during consumption of the low FODMAP diet (LFD, red) and moderate FODMAP diet (MFD, blue). a) Bacterial (16S rRNA) b) Fungal (ITS2) and c) MGS (shotgun metagenomic sequencing) profiles as identified by sparse partial least squares discriminant analysis (sPLS-DA). The plot represents the contribution of each operational taxonomic unit (OTU), whether obtained from 16S, ITS2 or MGS data, distinct between LFD and MFD with their contribution ranked from bottom (important) to top.


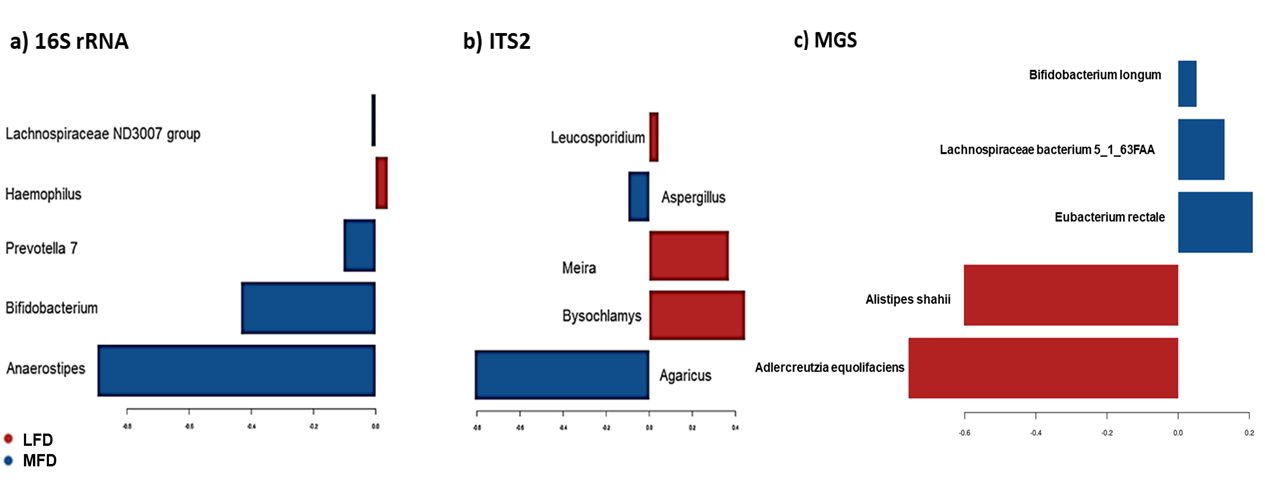


Supplementary Figure 3. Protein families (Pfam) differentiating the stool microbiota profiles of participants

during consumption of the low FODMAP (LFD; red) and moderate FODMAP (MFD; blue) diets identified by sPLS-DA. The plot represents the contribution of each feature (protein family category) with their contribution ranked from bottom (important) to top.


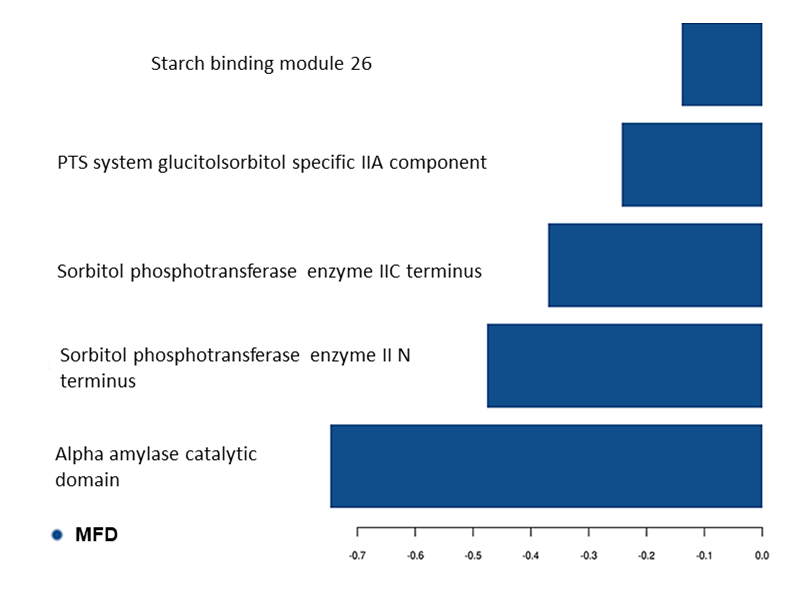


Supplementary Figure 4. The average nucleotide identity (ANI) matrix of the Bifidobacterial metagenome-assembled genomes (MAGs)

calculated from BLAST hits between orthologous genes of the core genome in FASTANI. The ANI scores calculated for the Bifidobacterial MAGs revealed 99.9% identity between bins recovered from the low FODMAP diet (LFD) group (LFD.119 and LFD.126) and the reference genome *B. animalis* subsp. *lactis* DSM 10140 suggesting these bins to be *B. animalis* strains. Reference genomes of *B. longum* NCC2705 and *B. adolescentis* ATCC 15703 were also included to build the ANI matrix. It revealed that bins recovered from the moderative FODMAP diet (MFD) group (MFD.81 and MFD.91) showed 98% and 97.8% identity respectively to the reference genome of *B. adolescentis* ATCC 15703 and bin MFD.92 had 98.43% identity to the reference genome of *B.longum NCC2705*.


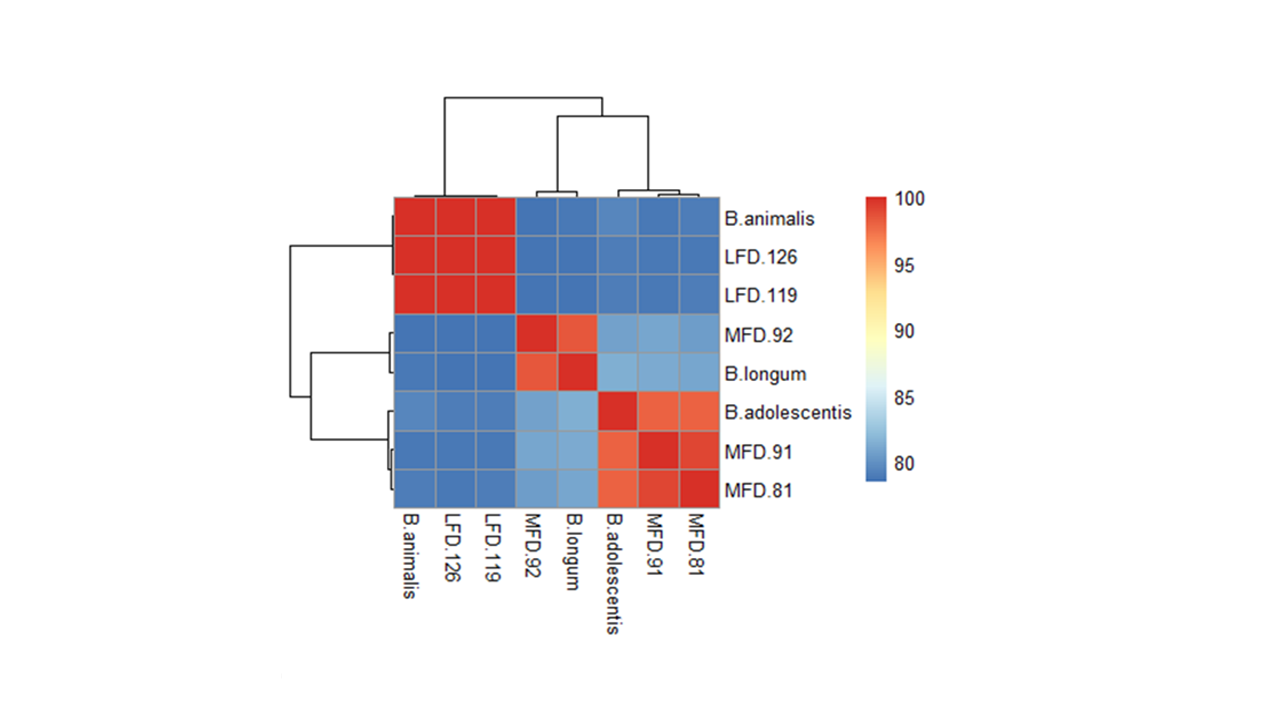


% nucleotide identity

**Supplementary Table 1.** Sample meal plan for one day of the low and moderate FODMAP diets, including the content of FODMAP oligosaccharides (‘oligos’ fructan + galacto-oligosaccharides) and polyols (sorbitol + mannitol). Foods written in italics represent the supplied dietary foods. Foods not written in italics were provided by the participants after they received detailed dietary instruction.

| Meal | Low FODMAP diet | Oligos (g) | Polyols  (g) | Moderate FODMAP diet | Oligos (g) | Polyols (g) |
| --- | --- | --- | --- | --- | --- | --- |
| **Breakfast** | *Pancakes (quinoa and rice flour), 1 serve*  Orange juice, 200 ml  Tea or coffee | *0.17*  *0.00* | *0.00*  *0.00* | *Pancakes ( lupin flour), 1 serve*  Apple juice, 200 ml  Tea or coffee | *0.48*  *0.00* | *0.00*  *0.55* |
| **Snack** | Banana, medium  Tub of reduced-fat yoghurt, 200 g  Tea, coffee or water | 0.52  0.00 | 0.00  0.00 | Apple, medium  Tub of reduced- fat yoghurt, 200 g  Tea, coffee or water | 0.00  0.00 | 1.54  0.00 |
| **Lunch** | *Sourdough spelt bread, 2 slices*  *Meatballs (no added onion or garlic), 1 serve*  *Capsicum dip (carrot, macadamia nuts and garlic-infused oil), 1 serve*  Reduced-fat cheese, 40 g  Lettuce, 1 cup  Tomato, 4 slices  Carrot, ½ cup grated  Tea, coffee or water | *0.24*  *0.00*  *0.00*  0.00  0.00  0.00  0.00 | *0.00*  *0.00*  *0.00*  *0.00*  *0.00*  *0.00*  *0.00* | *Wholemeal wheat bread, 2 slices*  *Meatballs (added onion, and garlic), 1 serve*  *Capsicum dip (sweet potato, almonds, onion, garlic), 1 serve*  Reduced-fat cheese, 40 g  Lettuce, 1 cup  Tomato, 4 slices  Carrot, ½ cup grated  Tea, coffee or water | *0.63*  *0.24*  *1.49*  0.00  0.00  0.00  0.00 | *0.00*  *0.00*  *0.08*  *0.00*  *0.00*  *0.00*  *0.00* |
| **Snack** | *Mixed nuts (peanuts, pecans, macadamia)*  Blueberries, 1 cup  Tea, coffee or water | *0.08*  *0.05* | *0.00*  *0.00* | *Mixed nuts (cashews, pistachios, hazelnuts)*  Apricot, 2 small  Tea, coffee or water | *0.85*  *0.22* | *0.00*  *1.37* |
| **Dinner** | *Lamb tagine (no added onion or garlic), 1 serve*  *Dukkah, 1 serve*  Quinoa, 1 cup cooked  Reduced-fat yoghurt, 2 tbsp.  Mint, 6 sprigs  Green beans, ¼ cup  Tea, coffee or water | *0.18*  *0.02*  0.06  0.00  0.00  0.00 | *0.16*  *0.00*  *0.00*  *0.00*  *0.00*  *0.08* | *Lamb tagine (added onion, garlic, mushrooms and kidney beans), 1 serve*  *Dukkah, 1 serve*  Couscous, 1 cup cooked  Reduced-fat yoghurt, 2 tbsp.  Mint, 6 sprigs  Sweet potato, ¼ cup  Tea, coffee or water | *2.98*  *0.02*  1.12  0.00  0.00  0.00 | *1.36*  *0.00*  *0.00*  *0.00*  *0.00*  *0.19* |
| **Total** |  | **1.32 g** | 0.48 g |  | **8.03 g** | **5.09g** |

**Supplementary Table 2.** The range of number of raw reads, filtered reads and % coverage of the MGS data according to diet.

| Diet group | Range (number of reads) before bowtie2 | Range (number of reads) after bowtie2 | Range % coverage |
| --- | --- | --- | --- |
| Low FODMAP (n=18) | 14.98-24.79 | 14.97-24.76 | 81-95.02 |
| Moderate FODMAP (n=18) | 15.54-25.71 | 15.51-25.68 | 80.6-94.6 |

|  |  |  |  |  |  |
| --- | --- | --- | --- | --- | --- |
| **Supplementary Table 3:** List of high-quality MAGs; >80% complete and <10% contaminated) extracted from the low and moderate FODMAP diet groups, taxonomically identified from hierarchical clustering using Mash in PATRIC | | | | | |
| **Bin Id** | **Marker lineage according to CheckM** | **Completeness** | **Contamination** | **Strain heterogeneity** | **Taxonomy as determined from PATRIC** |
| **Bins from low FODMAP diet group** | | | | | |
| final.contigs.fa.metabat-bins-.76 | p__Bacteroidetes (UID2605) | 91.06 | 2.08 | 80 | Alistipes obesi strain UBA9125 |
| final.contigs.fa.metabat-bins-.50 | p__Bacteroidetes (UID2605) | 80.33 | 9.09 | 27.78 | Alistipes onderdonkii strain S05A.meta.bin_1 |
| final.contigs.fa.metabat-bins-.53 | p__Bacteroidetes (UID2605) | 94.95 | 8.99 | 40 | Alistipes sp. CAG:268 |
| final.contigs.fa.metabat-bins-.119 | f__Bifidobacteriaceae (UID1460) | 99.56 | 4.36 | 0 | B. animalis |
| final.contigs.fa.metabat-bins-.126 | f__Bifidobacteriaceae (UID1460) | 99.33 | 3.41 | 94.12 | B.animalis |
| final.contigs.fa.metabat-bins-.30 | o__Bacteroidales (UID2657) | 91.18 | 6.39 | 42.86 | Bacteroides sp. CAG:98 |
| final.contigs.fa.metabat-bins-.124 | o__Clostridiales (UID1212) | 96.64 | 1.58 | 60 | Butyrivibrio crossotus strain 37_264 |
| final.contigs.fa.metabat-bins-.74 | k__Bacteria (UID2372) | 86.28 | 3.3 | 11.11 | Catenibacterium sp. AM22-15 |
| final.contigs.fa.metabat-bins-.114 | o__Clostridiales (UID1120) | 91 | 5 | 4.76 | Clostridiales bacterium strain UBA11500 |
| final.contigs.fa.metabat-bins-.110 | o__Clostridiales (UID1212) | 86 | 4.66 | 25 | Clostridium sp. CAG:352 |
| final.contigs.fa.metabat-bins-.92 | o__Clostridiales (UID1212) | 85.79 | 8.65 | 21.05 | Clostridium sp. GD3 |
| final.contigs.fa.metabat-bins-.68 | o__Clostridiales (UID1212) | 89.75 | 1.48 | 80 | Coprococcus sp. CAG:131 |
| final.contigs.fa.metabat-bins-.98 | o__Clostridiales (UID1212) | 83.22 | 1.57 | 100 | Eubacterium sp. CAG:38 |
| final.contigs.fa.metabat-bins-.130 | o__Clostridiales (UID1212) | 86.56 | 7.51 | 78.57 | Faecalibacterium prausnitzii strain S06A.meta.bin_6 |
| final.contigs.fa.metabat-bins-.127 | o__Clostridiales (UID1212) | 84.14 | 5.47 | 7.69 | Faecalibacterium sp. UBA2087 |
| final.contigs.fa.metabat-bins-.131 | o__Clostridiales (UID1212) | 86.07 | 5.06 | 19.05 | Firmicutes bacterium CAG:475 strain MGS:475 |
| final.contigs.fa.metabat-bins-.108 | o__Clostridiales (UID1226) | 85.44 | 6.03 | 58.33 | Lachnospiraceae bacterium strain UBA11783 |
| final.contigs.fa.metabat-bins-.166 | p__Proteobacteria (UID3887) | 83.12 | 7.43 | 77.78 | Parasutterella excrementihominis strain UBA9121 |
| final.contigs.fa.metabat-bins-.31 | f__Lachnospiraceae (UID1255) | 88.19 | 7.68 | 38.1 | Roseburia sp. CAG:100 |
| final.contigs.fa.metabat-bins-.89 | f__Lachnospiraceae (UID1286) | 82.9 | 4.2 | 40 | Roseburia sp. CAG:45 |
| final.contigs.fa.metabat-bins-.85 | o__Clostridiales (UID1212) | 88.59 | 9.68 | 19.35 | Ruminococcus sp. CAG:379 |
| final.contigs.fa.metabat-bins-.45 | o__Clostridiales (UID1226) | 87.14 | 6.86 | 56.25 | Ruminococcus sp. CAG:60 |
| final.contigs.fa.metabat-bins-.133 | g__Streptococcus (UID684) | 98.93 | 8.93 | 25 | Streptococcus thermophilus strain S9 |
| final.contigs.fa.metabat-bins-.75 | p__Proteobacteria (UID3887) | 98.14 | 2.88 | 14.29 | Sutterella sp. 63_29 |
| final.contigs.fa.metabat-bins-.62 | p__Bacteroidetes (UID2605) | 96.47 | 5.05 | 20 | uncultured Alistipes sp. strain UMGS657 |
| final.contigs.fa.metabat-bins-.146 | k__Bacteria (UID2565) | 95.7 | 3.32 | 75 | uncultured Azospirillum sp. strain UMGS148 |
| final.contigs.fa.metabat-bins-.49 | o__Bacteroidales (UID2617) | 86.16 | 5.11 | 25 | uncultured Bacteroidales bacterium strain UMGS440 |
| final.contigs.fa.metabat-bins-.78 | o__Clostridiales (UID1212) | 87.58 | 9.43 | 8.7 | uncultured Clostridiales bacterium strain UMGS1011 |
| final.contigs.fa.metabat-bins-.101 | o__Clostridiales (UID1212) | 88.43 | 2.36 | 14.29 | uncultured Clostridiales bacterium strain UMGS773 |
| final.contigs.fa.metabat-bins-.132 | o__Clostridiales (UID1120) | 88.96 | 3.32 | 33.33 | uncultured Clostridium sp. strain UMGS786 |
| final.contigs.fa.metabat-bins-.37 | o__Clostridiales (UID1212) | 96.71 | 5.85 | 35.29 | uncultured Eubacterium sp. strain UMGS173 |
| final.contigs.fa.metabat-bins-.35 | o__Clostridiales (UID1212) | 91.99 | 8.26 | 21.74 | uncultured Lachnospira sp. strain UMGS42 |
| final.contigs.fa.metabat-bins-.87 | o__Bacteroidales (UID2617) | 92.64 | 2.72 | 90.91 | uncultured Porphyromonadaceae bacterium strain UMGS49 |
| final.contigs.fa.metabat-bins-.47 | o__Bacteroidales (UID2716) | 86.52 | 8.06 | 3.45 | uncultured Prevotella sp. strain UMGS796 |
| final.contigs.fa.metabat-bins-.38 | o__Bacteroidales (UID2716) | 81.42 | 3.49 | 4.35 | uncultured Prevotella sp. strain UMGS937 |
| final.contigs.fa.metabat-bins-.42 | o__Clostridiales (UID1212) | 95.3 | 7.34 | 38.1 | uncultured Roseburia sp. strain UMGS1053 |
| final.contigs.fa.metabat-bins-.107 | o__Clostridiales (UID1212) | 83.09 | 6.85 | 13.64 | uncultured Ruminococcus sp. strain UMGS605 |
| final.contigs.fa.metabat-bins-.103 | o__Clostridiales (UID1212) | 84.3 | 6.94 | 64.29 | uncultured Ruminococcus sp. strain UMGS646 |
| **Bins from moderate FODMAP diet group** | | | | | |
| final.contigs.fa.metabat-bins-.123 | o__Clostridiales (UID1212) | 82.21 | 2.93 | 57.14 | [Eubacterium] siraeum strain MGYG-HGUT-02530 |
| final.contigs.fa.metabat-bins-.85 | p__Bacteroidetes (UID2605) | 89.68 | 2.64 | 25 | Alistipes obesi strain UBA9125 |
| final.contigs.fa.metabat-bins-.67 | p__Bacteroidetes (UID2605) | 94.47 | 5.2 | 52.94 | Alistipes sp. CAG:268 |
| final.contigs.fa.metabat-bins-.65 | p__Bacteroidetes (UID2605) | 91.72 | 8.27 | 34.62 | Alistipes sp. CAG:53 |
| final.contigs.fa.metabat-bins-.84 | p__Bacteroidetes (UID2605) | 93.65 | 2.59 | 20 | Alistipes sp. UBA1825 |
| final.contigs.fa.metabat-bins-.81 | f__Bifidobacteriaceae (UID1458) | 92.4 | 10.99 | 31.34 | B.adolescentis |
| final.contigs.fa.metabat-bins-.91 | f__Bifidobacteriaceae (UID1458) | 94.52 | 9.08 | 39.47 | B.adolescentis |
| final.contigs.fa.metabat-bins-.92 | f__Bifidobacteriaceae (UID1462) | 92.91 | 6.49 | 30.95 | B.longum |
| final.contigs.fa.metabat-bins-.98 | o__Clostridiales (UID1212) | 86.47 | 5.82 | 25 | Bacteroides pectinophilus CAG:437 |
| final.contigs.fa.metabat-bins-.30 | o__Bacteroidales (UID2657) | 90.3 | 1.87 | 36.36 | Bacteroides sp. CAG:98 |
| final.contigs.fa.metabat-bins-.28 | o__Bacteroidales (UID2657) | 95.77 | 5.07 | 6.9 | Bacteroides sp. Marseille-P3208T |
| final.contigs.fa.metabat-bins-.73 | o__Clostridiales (UID1120) | 91.24 | 8.04 | 0 | Clostridiales bacterium strain UBA11500 |
| final.contigs.fa.metabat-bins-.129 | o__Clostridiales (UID1212) | 95.97 | 5.99 | 26.67 | Clostridiales undefined |
| final.contigs.fa.metabat-bins-.121 | o__Clostridiales (UID1212) | 95.97 | 3.54 | 20 | Clostridiales undefined |
| final.contigs.fa.metabat-bins-.148 | o__Clostridiales (UID1212) | 82.57 | 0.74 | 0 | Clostridiales undefined |
| final.contigs.fa.metabat-bins-.49 | o__Clostridiales (UID1212) | 93.96 | 6.49 | 0 | Clostridium sp. CAG:352 |
| final.contigs.fa.metabat-bins-.53 | k__Bacteria (UID203) | 91.87 | 2.26 | 7.69 | Clostridium sp. CAG:510 |
| final.contigs.fa.metabat-bins-.34 | o__Clostridiales (UID1212) | 81.68 | 9.94 | 8.33 | Coprococcus eutactus strain MGYG-HGUT-00018 |
| final.contigs.fa.metabat-bins-.137 | o__Clostridiales (UID1212) | 82.9 | 4.01 | 81.82 | Coprococcus sp. CAG:131 |
| final.contigs.fa.metabat-bins-.90 | k__Bacteria (UID2372) | 96.23 | 2.36 | 0 | Erysipelotrichaceae bacterium strain MGYG-HGUT-00244 |
| final.contigs.fa.metabat-bins-.76 | k__Bacteria (UID203) | 91.23 | 9.17 | 16.67 | Eubacterium sp. CAG:38 |
| final.contigs.fa.metabat-bins-.105 | o__Clostridiales (UID1212) | 87.64 | 3.24 | 38.46 | Eubacterium sp. CAG:86 |
| final.contigs.fa.metabat-bins-.88 | o__Clostridiales (UID1212) | 92.98 | 3.16 | 63.64 | Eubacterium sp. CAG76_36_125 |
| final.contigs.fa.metabat-bins-.89 | o__Clostridiales (UID1212) | 81.84 | 8.8 | 69.57 | Firmicutes bacterium CAG:124 strain MGS:124 |
| final.contigs.fa.metabat-bins-.131 | o__Clostridiales (UID1212) | 86.8 | 5.67 | 7.69 | Firmicutes bacterium CAG:475 strain MGS:475 |
| final.contigs.fa.metabat-bins-.134 | k__Bacteria (UID2565) | 95.7 | 6.28 | 50 | Lactobacillus sp. N15.MGS-260 |
| final.contigs.fa.metabat-bins-.117 | f__Veillonellaceae (UID1032) | 93.27 | 2.42 | 14.29 | Mitsuokella sp. UBA4253 |
| final.contigs.fa.metabat-bins-.60 | o__Clostridiales (UID1226) | 94.62 | 8.33 | 0 | Oribacterium sp. strain W2P44.007 |
| final.contigs.fa.metabat-bins-.113 | o__Clostridiales (UID1212) | 90.67 | 8.11 | 14.29 | Oscillibacter sp. strain UBA11502 |
| final.contigs.fa.metabat-bins-.190 | p__Proteobacteria (UID3887) | 81.33 | 6.31 | 86.67 | Parasutterella excrementihominis strain UBA9121 |
| final.contigs.fa.metabat-bins-.139 | o__Selenomonadales (UID1024) | 90.62 | 3.55 | 54.55 | Phascolarctobacterium faecium DSM 14760 |
| final.contigs.fa.metabat-bins-.170 | k__Bacteria (UID2565) | 88.68 | 7.77 | 0 | Proteobacteria bacterium CAG:495 strain MGS:495 |
| final.contigs.fa.metabat-bins-.20 | f__Lachnospiraceae (UID1286) | 96.91 | 9.2 | 32.5 | Roseburia intestinalis CAG:13 |
| final.contigs.fa.metabat-bins-.64 | o__Clostridiales (UID1212) | 88.92 | 3.27 | 12.5 | Roseburia sp. CAG:380 |
| final.contigs.fa.metabat-bins-.174 | o__Clostridiales (UID1212) | 82.08 | 1.59 | 75 | Ruminococcaceae bacterium strain UBA9126 |
| final.contigs.fa.metabat-bins-.103 | f__Lachnospiraceae (UID1256) | 83.2 | 7.29 | 74.07 | Ruminococcus sp. CAG:55 |
| final.contigs.fa.metabat-bins-.63 | o__Clostridiales (UID1226) | 87.54 | 6.59 | 58.82 | Ruminococcus sp. CAG:60 |
| final.contigs.fa.metabat-bins-.100 | p__Proteobacteria (UID3887) | 91.25 | 6.64 | 15.79 | Sutterella sp. 63_29 |
| final.contigs.fa.metabat-bins-.33 | o__Clostridiales (UID1212) | 93.13 | 9.27 | 7.14 | uncultured Clostridiales bacterium strain UMGS22 |
| final.contigs.fa.metabat-bins-.165 | c__Clostridia (UID1118) | 84.53 | 7.82 | 27.78 | uncultured Clostridiales bacterium strain UMGS486 |
| final.contigs.fa.metabat-bins-.36 | o__Bacteroidales (UID2657) | 91.79 | 4.57 | 8.7 | uncultured Paraprevotella sp. strain UMGS1073 |
| final.contigs.fa.metabat-bins-.97 | o__Bacteroidales (UID2617) | 94.23 | 3.99 | 78.57 | uncultured Porphyromonadaceae bacterium strain UMGS49 |
| final.contigs.fa.metabat-bins-.80 | o__Bacteroidales (UID2617) | 93.02 | 4.21 | 0 | uncultured Porphyromonadaceae bacterium strain UMGS501 |
| final.contigs.fa.metabat-bins-.47 | o__Bacteroidales (UID2657) | 88.33 | 9.66 | 18.64 | uncultured Prevotella sp. strain UMGS796 |
| final.contigs.fa.metabat-bins-.111 | o__Clostridiales (UID1212) | 89.8 | 8.72 | 5.88 | uncultured Ruminococcus sp. strain UMGS1771 |
| final.contigs.fa.metabat-bins-.86 | o__Clostridiales (UID1212) | 93.9 | 3.75 | 57.14 | uncultured Ruminococcus sp. strain UMGS646 |

**Supplementary Table 4.** The gene counts of the enzymes known for carbohydrates and sugar alcohol degradation in the five recovered Bifidobacteria metagenome assembled genomes (MAGs).

| **Carbohydrate degrading enzymes (oligosaccharides and polyols)** | LFD.119 | LFD.126 | MFD.91 | MFD.81 | MFD.92 |
| --- | --- | --- | --- | --- | --- |
| Alpha-mannosidase | 0 | 0 | 5 | 3 | 2 |
| Beta-galactosidase | 5 | 4 | 18 | 12 | 16 |
| Beta-glucosidase | 4 | 3 | 9 | 25 | 12 |
| Sorbitol dehydrogenase | 0 | 0 | 0 | 0 | 1 |
| Transcriptional regulator of various polyols utilisation, AraC family | 0 | 0 | 1 | 1 | 1 |
